# Synthesis of a novel polycyclic ring scaffold with antimitotic properties *via* a selective domino Heck–Suzuki reaction†
†Electronic supplementary information (ESI) available: Full experimental details, ^1^H and ^13^C NMR spectra and X-ray crystallographic data for compound **4d**. CCDC 936207. For ESI and crystallographic data in CIF or other electronic format see DOI: 10.1039/c4sc02547d



**DOI:** 10.1039/c4sc02547d

**Published:** 2014-09-09

**Authors:** Esther Alza, Luca Laraia, Brett M. Ibbeson, Súil Collins, Warren R. J. D. Galloway, Jamie E. Stokes, Ashok R. Venkitaraman, David R. Spring

**Affiliations:** a Department of Chemistry , University of Cambridge , Lensfield Road , Cambridge , CB2 1EW , UK . Email: spring@ch.cam.ac.uk ; Fax: +44 (0)1223 336362 ; Tel: +44 (0)1223 336498; b MRC Cancer Unit , University of Cambridge , Hutchison/MRC Research Centre , Biomedical Campus , Hills Road , Cambridge , CB2 0XZ , UK

## Abstract

The synthesis of a previously undescribed sp^3^-rich 6-5-5-6 tetracyclic ring scaffold using a palladium catalysed domino Heck–Suzuki reaction is reported. This reaction is high-yielding, selective for the domino process over the direct Suzuki reaction and tolerant towards a variety of boronic acids. The novel scaffold can also be accessed *via* domino Heck–Stille and radical cyclisations. Compounds based around this scaffold were found to be effective antimitotic agents in a human cancer cell line. Detailed phenotypic profiling showed that the compounds affected the congression of chromosomes to give mitotic arrest and apoptotic cell death. Thus, a novel structural class of antimitotic agents that does not disrupt the tubulin network has been identified.

## Abstract 2

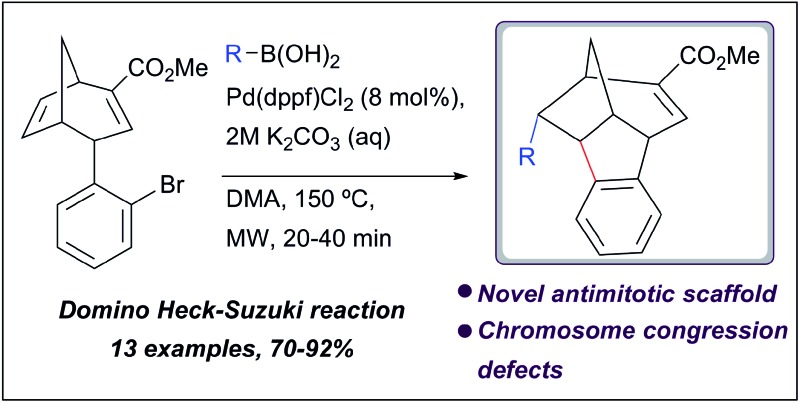
The synthesis of a previously undescribed sp^3^-rich 6-5-5-6 tetracyclic ring scaffold using a palladium catalysed domino Heck–Suzuki reaction is reported.

## Introduction

The molecular scaffold of a small molecule can be considered as its core rigidifying structural feature.^1^ Of all the structural elements of a small molecule, it is the scaffold that has the largest impact upon its overall shape, and thus how it displays its chemical information (*i.e.* functional groups and potential binding motifs) in three-dimensions (3D).^2^ The scaffold of a small molecule is therefore a key determinant of its biological activity, since a given biological macromolecule will only interact with those small molecules that have a complementary 3D binding surface.^1b,2a,3,4^ However, the known universe of organic chemistry is dominated by a strikingly small percentage of molecular scaffolds.^2a,b,5^ Unsurprisingly therefore, recent years have witnessed considerable interest in the development of new synthetic strategies to access novel molecule scaffolds that have yet to be exploited in a therapeutic context;^6^ such scaffolds may be associated with unusual and useful biological properties (*e.g.*, activity against a previously unexploited target/process or a novel mode of action), as well as offering access to novel intellectual property.^2a,b,5b,c^ In particular, there is a demand for new molecular scaffolds with high levels of three-dimensionality, (that is, a high sp^3^ carbon count), as this has been shown to correlate positively with reduced clinical attrition of candidate drugs (and reduced toxicity).^7^

Access to novel molecular scaffolds may prove valuable in a number of therapeutic areas. One such area is the treatment of cancer using small molecules that interfere with the normal progression of mitosis (antimitotics). Antimitotic compounds have been used clinically for decades and this target class is widely regarded to still hold great potential for anti-cancer therapy.^8,9^ All currently approved antimitotic compounds target tubulin.^8,10^ However, increased resistance and administration problems suggest that new structural classes of antimitotic compounds are required, perhaps even with novel modes of action.^8,10a,11^ Indeed, recent years have witnessed considerable efforts directed towards the discovery of new antimitotic compounds that could offer an alternative to existing therapies.^8,12^

**Scheme 1:**
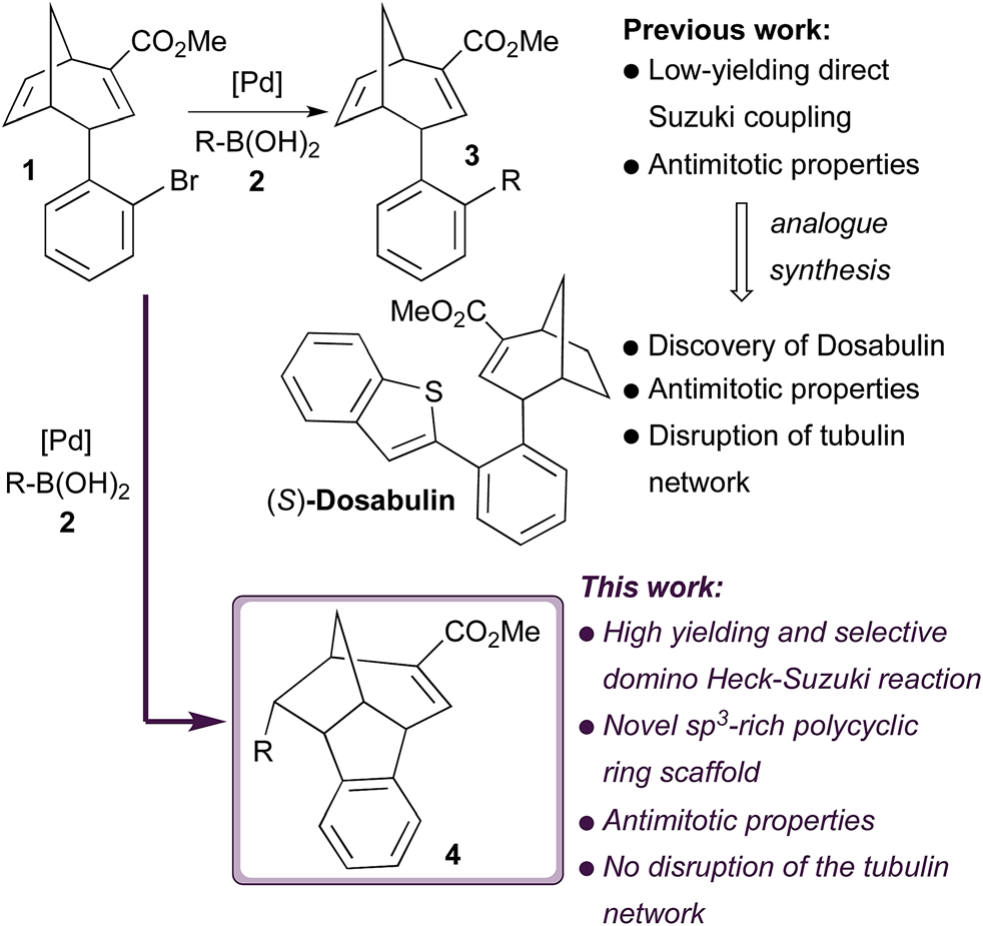
Synthesis of novel 6-5-5-6 tetracyclic ring scaffold **4**.

Recently, we identified a series of antimitotics which disrupted the tubulin network using a high-content image based screening (HCS) of a diversity-oriented synthesis (DOS) library.^8a,13^ These compounds were synthesised by Suzuki coupling of densely functionalised bicyclo[3.2.1]octadiene **1** with boronic acids **2** and were based around scaffold **3** (Scheme 1).

Analogue synthesis and biological evolution led to the discovery of Dosabulin, a structurally novel antimitotic agent that disrupts the tubulin network by acting as a tubulin depolymerisation agent. The yields for the Suzuki coupling process leading to compounds **3** were surprisingly low, which prompted us to investigate this reaction further. Herein, we describe the identification of a competing reaction pathway involving a palladium catalysed domino Heck–Suzuki reaction that leads to the novel, complex and sp^3^-rich scaffold **4**, which contains a 6-5-5-6 bridged and fused ring system (Scheme 1). Two new stereocentres are formed with complete diastereocontrol in this domino reaction sequence. Optimisation studies led to reaction conditions that are completely selective for the domino process over the direct Suzuki reaction. A small library of compounds based around unprecedented scaffold **4** was generated. Phenotypic screening of this library for induction of mitotic arrest identified several hits in the low micromolar range and further analysis revealed that they are capable of interfering with the correct congression of chromosomes to give cell death by apoptosis. Significantly, there was no evidence that the active compounds disrupt the tubulin network, unlike active antimitotics based around scaffold **3**, Dosabulin^8*a*^ and all mitotic inhibitors currently approved for clinical use; antimitotics that do not target tubulin are highly sought after.^11b,c^ Scaffold **4** may thus define a new structural class of antimitotic with an unusual (or perhaps even novel) mode of action.

## Results and discussion

The synthesis of **1** was achieved *via* rhodium(ii)-catalysed carbenoid chemistry.^8*a*^ This compound was purposely designed to contain an *ortho*-bromo aryl functionality in order to allow diversification through transition-metal catalysed reactions. Under standard Suzuki conditions, **1** reacted with boronic acid **2a** to yield the biaryl product **3a** in poor yield (Table 1, entry 1).^8*a*^ Isolation and analysis of the main side product resulted in the identification of a competing reaction pathway involving an intramolecular Heck reaction followed by an intermolecular Suzuki reaction, leading to compound **4a**. This structurally complex 6-5-5-6 polycyclic ring scaffold was obtained in one step with the concomitant formation of two new stereocentres. Domino (cascade) reactions have been extensively used in small molecule library synthesis,^14^ incorporating multiple synthetic sequences to rapidly yield structurally complex products (often based around complex 3D scaffolds). Domino Heck–Suzuki reactions have been previously reported for the synthesis of isoindolinones^15^ and combretastatin^16^ analogues among others,^17^ however no examples of sp^3^–sp^2^ couplings in the Suzuki step have been reported.

**Table 1:** Investigation of reaction conditions^*a*^

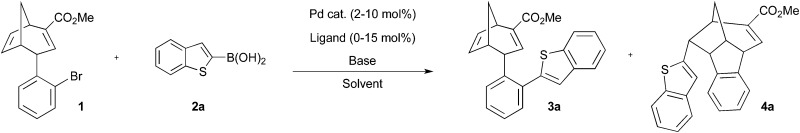								
Entry	Pd source	Ligand	Solvent	Base	Temp (°C)	Time	Ratio (**3a** : **4a**)	Yield^*b*^ (%)
**1**	Pd(OAc)_2_ (10 mol%)	PPh_3_ (15 mol%)	Toluene	K_2_CO_3_ (2 M aq.)	90	12 h	1 : 2	51 (**4a**)
**2**	Pd(OAc)_2_ (10 mol%)	P^*t*^Bu_3_ (15 mol%)	Toluene	K_2_CO_3_ (2 M aq.)	90	12 h	50 : 1	70 (**3a**)
**3**	Pd(OAc)_2_ (10 mol%)	PhDavePhos (10 mol%)	THF	K_3_PO_4_	rt	12 h	—	n.r.^*c*^
**4**	Pd(OAc)_2_ (10 mol%)	XPhos (10 mol%)	THF	K_3_PO_4_	rt	12 h	—	n.r.^*c*^
**5**	Pd(OAc)_2_ (10 mol%)	—	Toluene	K_2_CO_3_ (2 M aq.)	90	12 h	>100 : 1	25 (**3a**)
**6**	Pd(OAc)_2_ (10 mol%)	—	Toluene	CsF	90	12 h	>100 : 1	65 (**3a**)
**7**	Pd(PPh_3_)_4_ (5 mol%)	—	Toluene	K_2_CO_3_ (2 M aq.)	90	5 h	3 : 1	23 (**3a**)
**8**	Pd(PPh_3_)_4_ (5 mol%)	—	Toluene	CsF	90	5 h	1 : 9	<10 (**4a**)
**9**	Pd(PPh_3_)_4_ (5 mol%)	—	Toluene	Cs_2_CO_3_	90	5 h	4 : 1	36 (**3a**)
**10**	Pd(OAc)_2_ (10 mol%)	—	1,4-Dioxane	K_2_CO_3_	90	12 h	>100 : 1	77 (**3a**)
**11**	Pd(OAc)_2_ (10 mol%)	—	1,4-Dioxane	CsF	90	12 h	>100 : 1	78 (**3a**)
**12**	Pd(PPh_3_)_4_ (5 mol%)	—	1,4-Dioxane	K_2_CO_3_ (2 M aq.)	90	5 h	3 : 1	58 (**3a**)
**13**	Pd(PPh_3_)_4_ (5 mol%)	—	1,4-Dioxane	CsF	90	5 h	1 : 9	<10 (**4a**)
**14**	Pd(PPh_3_)_4_ (5 mol%)	—	1,4-Dioxane	Na_2_CO_3_ (2 M aq.)	90	5 h	6 : 1	70 (**3a**)
**15**	Pd(PPh_3_)_4_ (5 mol%)	—	1,4-Dioxane	Cs_2_CO_3_	90	5 h	3 : 1	48 (**3a**)
**16**	Pd(OAc)_2_ (10 mol%)	—	1,4-Dioxane	CsF	100^*f*^	1.5 h	>100 : 1	81 (**3a**)
**17** ^*d*^	Pd(OAc)_2_ (10 mol%)	—	1,4-Dioxane	CsF	130^*f*^	0.5 h	>100 : 1	80 (**3a**)
**18** ^*e*^	Pd(OAc)_2_ (2 mol%)	—	1,4-Dioxane	CsF	130^*f*^	1.5 h	>100 : 1	83 (**3a**)
**19** ^*d*^	Pd(PPh_3_)_4_ (5 mol%)	—	1,4-Dioxane	K_2_CO_3_ (2 M aq.)	130^*f*^	1.5 h	1 : >100	87 (**4a**)
**20**	Pd(dppf)Cl_2_ (8 mol%)	—	DMA	K_2_CO_3_ (2 M aq.)	150^*f*^	0.5 h	1 : >100	92 (**4a**)

^*a*^All the reactions were carried out with 3 equivalents **2a** and 3 equivalents of base (unless otherwise stated), under the corresponding conditions showed for each reaction. Thermal irradiation was used unless otherwise indicated.

^*b*^Yield of isolated major products.

^*c*^No reaction (n.r.).

^*d*^2 equivalents **2a** were added.

^*e*^1.5 equivalents **2a** were added.

^*f*^Reaction heated in sealed tube under microwave irradiation.

We were interested in identifying conditions that could selectively deliver either **3** or **4**. The use of a bulkier ligand (Table 1, entry 2), resulted in a switch in the selectivity towards the formation of **3a**, which was isolated in moderate yield. In an attempt to increase this selectivity and yield, conditions developed by Buchwald^18^ using lower temperatures and bulkier ligands were explored. Unfortunately, no reaction was observed, with all of the starting material being recovered (Table 1, entries 3 and 4). On the other hand, when the reaction was carried out in the absence of ligand, **3a** was obtained exclusively, and the yield was improved by the use of CsF as base (Table 1, entries 5 and 6 respectively). The use of a pre-formed palladium complex such as Pd(PPh_3_)_4_, led to mixtures of both possible products in differing ratios depending on the base added (Table 1, entries 7–9 and 12–15). Among the solvents tested, 1,4-dioxane was the most efficient for the formation of the direct Suzuki product **3a**. High selectivity for this compound was observed when Pd(OAc)_2_ was used as catalyst (Table 1, entries 10 and 11). In an attempt to increase the reaction rate, the temperature was increased and reactions were performed under microwave irradiation. These new conditions provided **3a** in good yields and with excellent levels of selectivity after only relatively short reaction times (Table 1, entries 16 and 17), even with catalyst loadings of just 2 mol% and 1.5 equivalents of boronic acid (Table 1, entry 18). Pleasingly, variation of the palladium source and base (Table 1, entry 19) led to the highly selective formation of the domino Heck–Suzuki product **4a**, which could be isolated in a high yield. The reaction rate and isolated yield of **4a** could be increased further through the use of bidentate phosphine ligand 1,1′-bis(diphenylphosphino)ferrocene (dppf) in combination with a slightly higher temperature and DMA as the solvent (Table 1, entry 20).

**Scheme 2:**
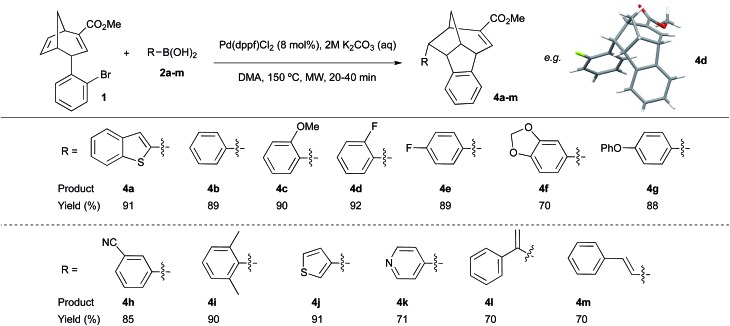
Domino Heck–Suzuki reactions of boronic acids **2a–m** with bicyclo[3.2.1]octadiene **1** to form compounds **4a–m**. MW = microwave irradiation. 2 equivalents of boronic coupling partner (**2a–m**) were used in all reactions. Yields of isolated products given in all cases. X-ray crystal structure of compound **4d** shown.

To explore the scope of both the direct Suzuki and domino Heck–Suzuki reactions of **1**, several aryl, *S*- and *N*-heteroaryl, as well as vinyl boronic coupling partners (**2a–m**, Scheme 2) were tested under the optimised conditions for the selective formation of **3a** and **4a** respectively. Unexpectedly, under the optimised conditions for the direct Suzuki coupling of **1** with **2a** (Table 1, entry 17) only 4-phenoxyphenyl boronic acid **2g** produced the direct Suzuki cross-coupling product **3g** in 72% yield (data not shown). Reaction of **1** with all other boronic acids under these conditions yielded recovered starting material. In contrast, under reaction conditions optimised for the domino Heck–Suzuki process (Table 1, entry 20) high yields and complete selectivity for compounds **4a–m** were observed for all the boronic acids tested, with reaction times ranging from 20–40 minutes (Scheme 2). These included traditionally difficult sp^3^–sp^2^ carbon Suzuki couplings (Scheme 2, **4l** and **4m**). An X-ray crystal structure of compound **4d** was obtained (Scheme 2), which confirmed the relative stereochemistry of the new chiral centres generated during the domino reaction process.

**Scheme 3:**
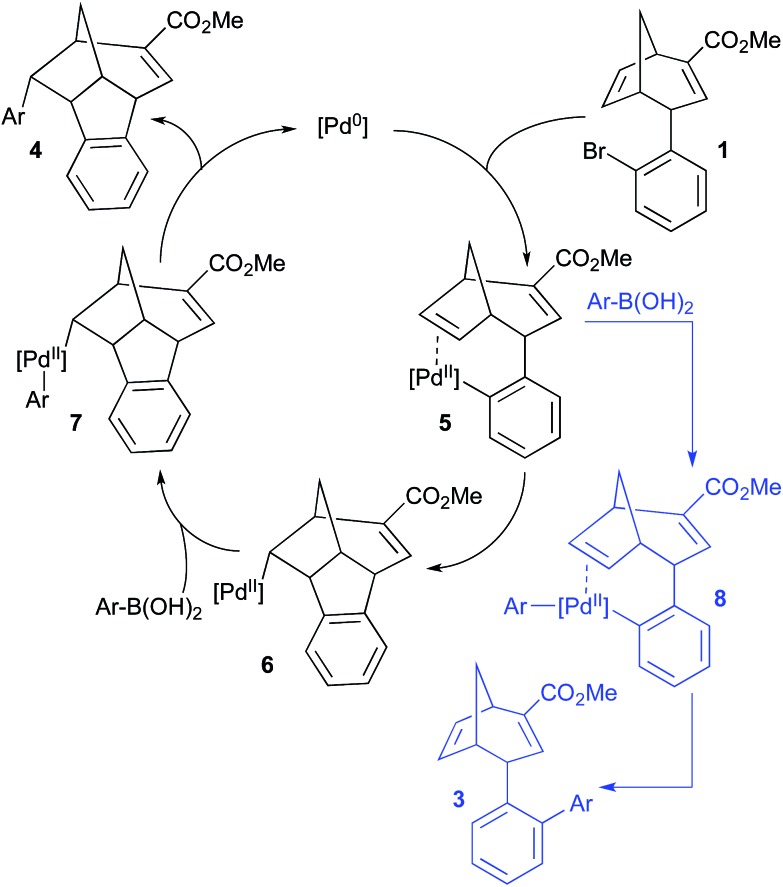
Proposed reaction mechanism pathways for the domino Heck–Suzuki process (black) and the direct Suzuki reaction (competing reaction pathway shown in blue).

A tentative mechanistic hypothesis for the domino Heck–Suzuki reaction is outlined in Scheme 3. Initial oxidative addition of the aryl bromide **1** to the Pd(0) species to form **5** is followed by a *syn*-migratory insertion across the adjacent double bond to deliver intermediate **6**. The plausibility of this sequence is supported by research carried out by Catellani and co-workers, who have shown that the alkene of the structurally related compound norbornene reacts readily with ArPdX species.^19^ The observed selectivity for reaction of the more strained alkene of intermediate **5**, in preference to the electron deficient alkene that is embedded within a larger ring, is not unexpected. *β*-hydride elimination of the adjacent bridgehead proton of **6** would result in the formation of a double bond with one terminus at the bridgehead of the fused ring system; thus, this process would be expected to be strongly disfavoured. Consequently, transmetalation of **6** with the boronic coupling partner is likely to be preferred, which would lead to species **7**. Subsequent reductive elimination would then furnish the domino Heck–Suzuki product **4**. The selectivity for the domino product over the direct Suzuki product may be a consequence of alkene insertion (converting **5** to **6**) occurring significantly faster than the competing transmetalation to form **8** (which subsequently could undergo reductive elimination to form **3**); this could possibly be due to the close proximity of the alkene and Pd in **5**, the relief of ring strain upon insertion into the alkene, or as a result of steric effects impeding the approach of the boronic acid. However, it is not readily apparent how variation in the conditions should affect the relative rates of these two pathways and thus the reaction outcome; a complex interplay of factors appears to be involved.^20^ Furthermore, we are not able to explain the lack of reactivity in the direct Suzuki reaction for most of the boronic acids (however, we hypothesize that the benzothiophene and biaryl ether substrates **2a** and **2g** enhance the reactivity of an otherwise unreactive system). Clearly, further and more detailed mechanistic studies are required in order to more fully delineate the progress of the domino Heck–Suzuki reaction and thus better rationalise the basis of the observed selectivity over the competing direct Suzuki coupling pathway.

**Scheme 4:**
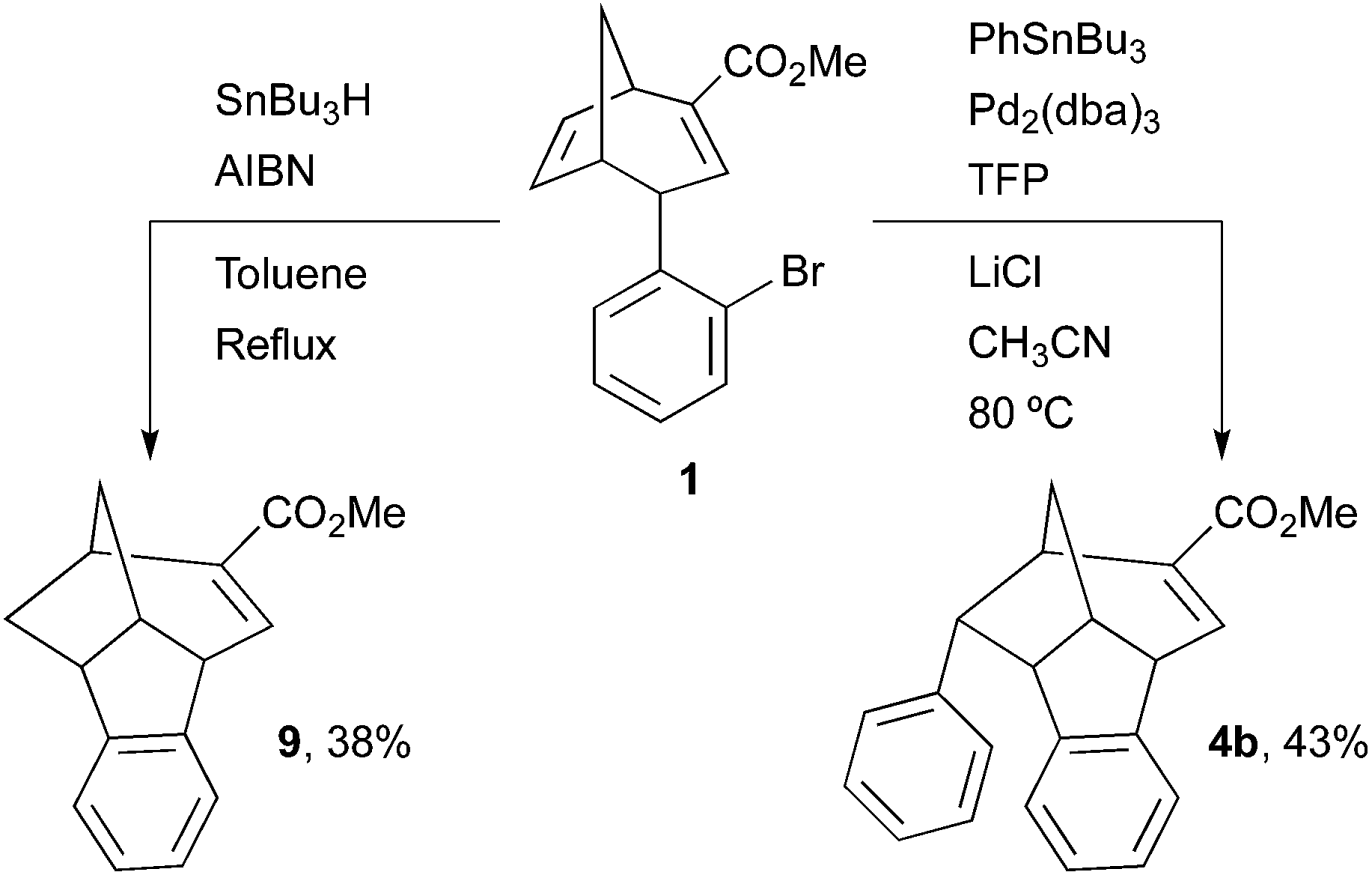
Alternative domino Heck–Stille and radical cyclisations. TFP = tri-(2-furyl)phosphine.

In addition to the domino Heck–Suzuki reaction, we envisaged that the 6-5-5-6 tetracyclic ring system could be formed by complementary domino cyclisations of **1**. For example, the use of tributylphenylstannane instead of a boronic acid afforded compound **4b** in an unoptimised yield of 43% *via* a domino Heck–Stille mechanism (Scheme 4).^21^ Interestingly, no biaryl product resulting from the direct Stille reaction was observed. This is a promising result for an unoptimised process, and it also suggests that the selectivity of the domino Heck–Stille reaction is governed by similar factors to those controlling the outcome of the domino Heck–Suzuki reaction. Optimisation studies may allow an increase in the yield of **4b** that can be obtained *via* the domino Heck–Stille process, and variation of the organostannane coupling partner may be tolerated, potentially allowing the introduction of novel substituents on the core scaffold. This represents an on-going area of work and results will be reported in due course. The synthesis of compound **9**, based on the core ring system of **4** but without an aromatic substituent present, was also desired, as it was thought that this would provide an interesting point of comparison in any structure–activity relationship studies. This was achieved in a low (unoptimised) yield by a tin radical catalysed cyclisation process (Scheme 4).^22^

**Fig. 1:**
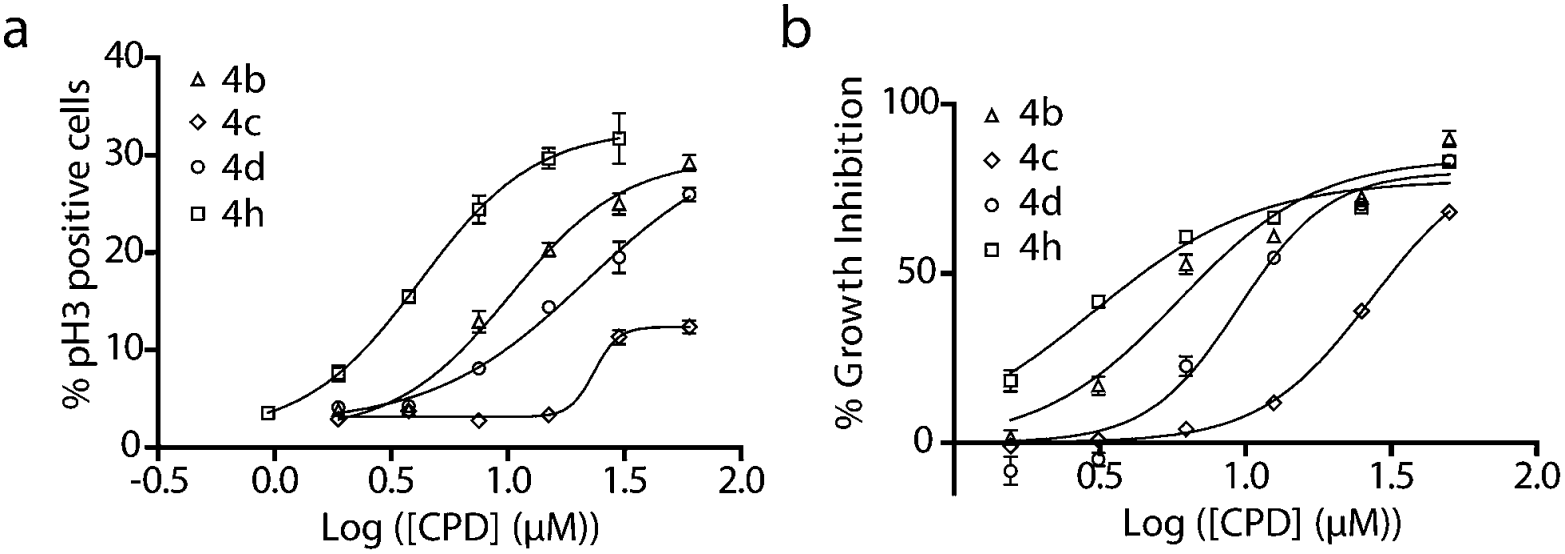
Antimitotic and growth inhibitory effects of fused ring analogues **4**: (a) mitotic arrest induced by **4b–d**, and **4h**; U2OS cells were incubated with compounds for 20 h before being fixed and stained with Hoechst (a DNA dye) and a pH3 specific antibody. Cells were imaged and analysed on a Cellomics Arrayscan. Data shown as the mean ± sem for an experiment conducted in triplicate. CPD = compound; (b) Growth inhibition of U2OS cells by **4b–d**, and **4h**. U2OS cells were incubated with compounds for 72 h before fixing and staining with sulforhodamine B. Absorbance was then measured at 510 nm. Data is shown as the mean ± sem for an experiment conducted in triplicate.

All compounds based around the novel 6-5-5-6 tetracyclic ring scaffold were tested for their ability to induce mitotic arrest in human osteosarcoma cells (U2OS). This was done using a high content screening (HCS) approach, where cells are stained with an antibody against the mitotic marker phospho-Histone H3 (pH3) and imaged on a Cellomics Arrayscan high content microscope.^8^ An automated image analysis algorithm then calculated the percentage of mitotic cells. Several compounds displayed a significant dose-dependent increase in mitotic cells (Fig. 1a and Table 2) with EC_50_ values in the low micromolar range. Preliminary structure–activity relationships suggest that *ortho* and *meta* substituents are tolerated, whilst *para* substituents are not. Compound **9**, which does not contain an aromatic substituent around the core scaffold, was inactive. The most potent compound was the *meta-*cyano analogue **4h** with an EC_50_ of 4.8 μM. The thiophene containing analogue **4j** retained very weak activity, whilst pyridine containing analogue **4k** and vinyl substituted compound **4l** were inactive (MI EC_50_ > 100 μM). All antimitotic compounds were also able to inhibit the growth of the same cancer cell line at low micromolar concentrations (as determined by a 72 hour sulforhodamine B assay for cytotoxic effects against the U2OS cell line, Fig. 1b).

**Table 2:** Antimitotic and growth inhibitory activity of domino Heck–Suzuki reaction products in U2OS cells^*a*^

Product	Mitotic index (MI) EC_50_ (μM)	Growth inhibition GI_50_ (μM)
**4b**	19.1 ± 3.5	8.40 ± 3.5
**4c**	>25	>25
**4d**	13.6 ± 1.2	8.40 ± 1.6
**4h**	4.80 ± 2.1	3.90 ± 1.2
**4j**	30.8 ± 2.7	22.3 ± 1.9

^*a*^All data is the mean ± SD of three experiments conducted in triplicate.

**Fig. 2:**
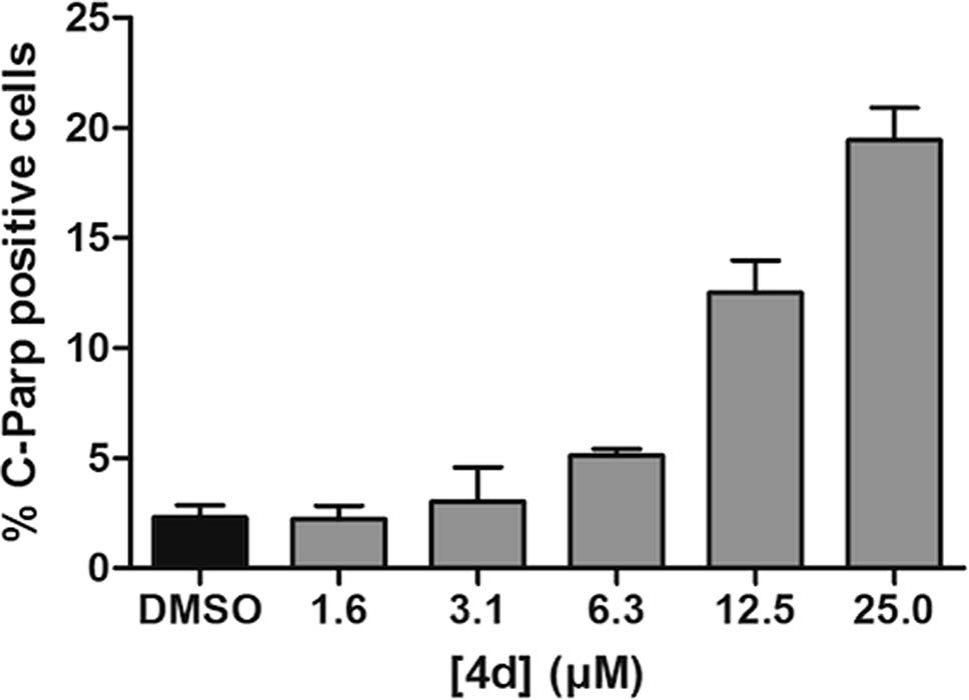
Induction of apoptosis by **4d**. U2OS cells were incubated with **4d** for 72 h before fixing and staining with Hoechst and a cleaved PARP specific antibody. Cells were imaged and analysed on a Cellomics Arrayscan. Data shown as the mean ± sem for an experiment conducted in triplicate.

To determine whether apoptosis was the mechanism of cell death another HCS with cleaved PARP as the apoptotic marker was used (Fig. 2).^23^ This showed that cells exposed to the representative antimitotic **4d** underwent apoptosis at concentrations similar to those required for the induction of mitotic arrest and growth inhibition, suggesting that mitotic arrest is followed by cell death through apoptosis.

**Fig. 3:**
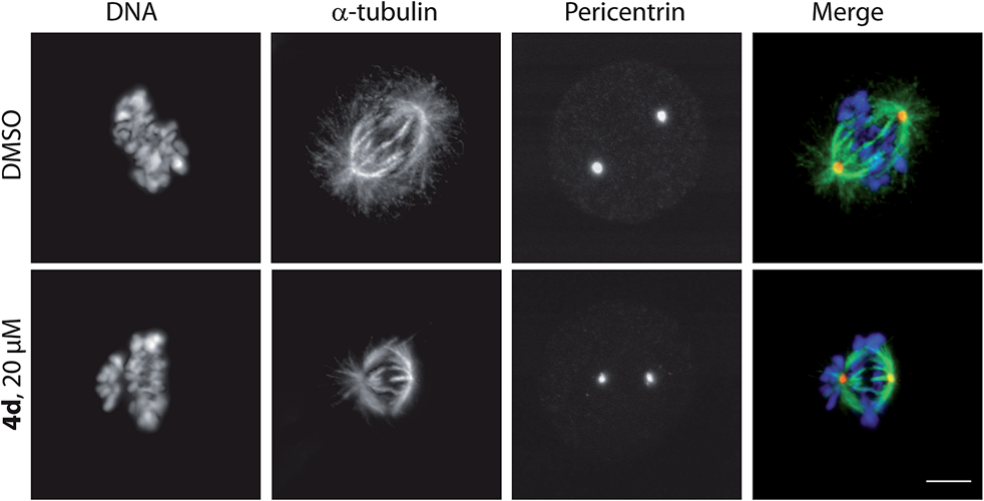
Analysis of mitotic phenotype of U2OS cells with 20 μM **4d** shows chromosome congression defects. Cells were treated with **4d** for 20 h before being fixed and stained with DAPI (blue), α-tubulin (green) and pericentrin (red); scale bar = 6 μm.

**Fig. 4:**
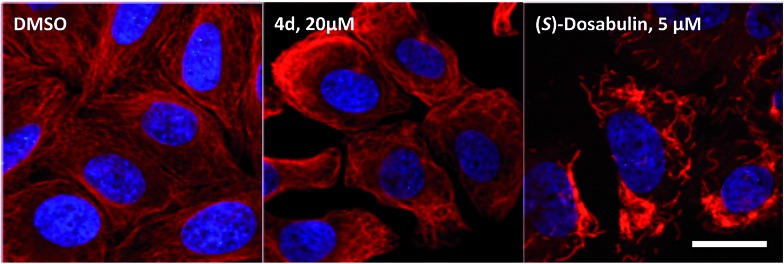
Analysis of interphase U2OS cells treated with 20 μM **4d** or DMSO (control) shows no difference in the tubulin network. Cells treated with 5 μM (*S*)-Dosabulin show a clear disruption in the tubulin network. Cells were treated with **4d** or (*S*)-Dosabulin for 20 h before being fixed and stained with DAPI (blue), α-tubulin (red) and pericentrin (green). Scale bar = 10 μm.

To study the phenotype of mitotic arrest further, cells were imaged at high magnification using a confocal microscope (Fig. 3). This showed that cells treated with **4d** displayed a non-congressed chromosome phenotype compared to the DMSO treated control. Importantly, the tubulin network was not affected in interphase cells (Fig. 4), suggesting that these compounds act by a different mechanism than the active compounds based around scaffold **3** and Dosabulin, which clearly disrupt the tubulin network.^8*a*^ It is interesting to note that two closely related scaffolds (**3** and **4**) show distinct modes-of-action leading to mitotic arrest. Comparison of the X-ray crystal structure obtained for **4d** (Scheme 2) with that of (*R*)-Dosabulin^8*a*^ reveals pronounced differences in their 3D shape. Thus, although the resulting differences in mode-of-action may not be surprising, the common mitotic arrest certainly is. It is a testament to the strength of the DOS approach that two diverse sp^3^ rich scaffolds derived from the same intermediate are biologically relevant with different modes of action.^6^

## Conclusions

In conclusion, we have described a route to a previously unreported sp^3^-rich 6-5-5-6 tetracyclic ring scaffold using a palladium catalysed domino Heck–Suzuki reaction. Reactions proceed rapidly, selectively and in high yield for a large range of boronic acids. The selectivity could be completely reversed to deliver the direct Suzuki product, however this was found to occur for specific boronic acids only. More detailed mechanistic studies into the domino reaction process are ongoing and results will be reported in due course. In addition to domino Heck–Suzuki reactions, domino Heck–Stille and radical cyclisations delivered the same polycyclic ring scaffold, expanding the available routes to access such compounds. Compounds based around this novel scaffold were shown to possess antimitotic activity against human osteosarcoma cells (U2OS line), which resulted in cancer cell death *via* an apoptotic mechanism. Thus, a new structural class of antimitotic agents has been identified. Detailed phenotypic profiling showed that the compounds caused chromosome congression defects, with DNA failing to align at the metaphase plate. Significantly, there was no evidence that the active compounds disrupt the tubulin network, unlike the antimitotics based around scaffold **3** and Dosabulin (identified in our previous study^8*a*^) and all mitotic inhibitors currently approved for clinical use. Thus, scaffold **4** may define a new structural class of antimitotic with an unusual (or perhaps even novel) mode of action, though further investigations are required in this area. Efforts towards identifying the targets of the antimitotic compounds described will be reported in due course. This work highlights the potential of domino reactions to deliver structurally complex biologically relevant small molecules. The methodology reported could potentially be applied to similar ring systems (such as norbornenes or bicyclo[2.2.2]octenes), which may offer access to further novel and stereochemically rich scaffolds.
