# Supplementary material for: Synthesis of a novel polycyclic ring scaffold with antimitotic properties via a selective domino Heck–Suzuki reaction
Source: Chem Sci. 2014 Sep 9;6(1):390–6. doi: 10.1039/c4sc02547d (PMC5586250; doi:10.1039/c4sc02547d)
Supplement: Supplementary file 1 [file SC-006-C4SC02547D-s001.pdf]

Electronic Supplementary Material (ESI) for Chemical Science.
This journal is © The Royal Society of Chemistry 2014

Supplementary Information

Synthesis of a Novel Polycyclic Ring Scaffold with Antimitotic Properties via a Selective Domino Heck-Suzuki reaction

Esther Alza, Luca Laraia, Brett M. Ibbeson, Súil Collins, Warren R. J. D. Galloway, Jamie E. Stokes, Ashok R. Venkitaraman, David R. Spring*

General Experimental Details 4
General Procedure 1 : Domino Heck-Suzuki Coupling using Pd(dppf)Cl_2 6
Compound Preparation and Characterisation 7
NMR Spectra of Novel Compounds: 19
X-ray Crystallographic Data: 31
Biological Methods: 40
  High Content Analysis – Mitotic Index 40
  Sulforhodamine B colorimetric assay for cytotoxicity screening 40
  Confocal Microscopy 41
  High Content Analysis – Apoptosis: 41

General Experimental Details

Chemicals were purchased from Sigma Aldrich or Alfa Aesar and were used as received unless otherwise stated. Room temperature refers to ambient temperatures of 20–25 °C. All solvents were anhydrous unless otherwise stated.

Where possible, reactions were monitored by thin layer chromatography (TLC) perfomed on commercially prepared Merck Kieselgel 60 F254 plates. Visualisation was by the quenching of ultraviolet light (ν_{max} = 254 nm) or by staining with potassium permanganate. Retention factors (R_f) are given to 0.01. Flash column chromatography was performed using Sigma Aldrich 60 (230–240) mesh silica columns under a positive pressure of nitrogen.

Infra-red spectra were recorded on a Perkin Elmer Spectrum One FT-IR spectrometer. Maximum absorbance (ν_{max}) of selected peaks are given in wavenumbers (cm^{-1}) and the absorbance intensity described as broad (br), weak (w), medium (m) or strong (s) with the assignment given after.

Proton nuclear magnetic resonance (^{1}H NMR) and carbon-13 nuclear magnetic resonance (^{13}C NMR) were recorded at 300 K on a Bruker Avance III 400 spectrometer and a Bruker DPX 500 spectrometer. Chemical shifts (δ) are quoted in ppm relative to the residual non-deuterated solvent peak and coupling constants (*J*) are quoted to the nearest 0.5 Hertz (Hz). Spectral data is reported as follows: chemical shift, integration, multiplicity [s, singlet; d, doublet; t, triplet; app t, apparent triplet; m, multiplet], coupling constant(s) and assignment. Assignments are supported by APT, DEPT, COSY, HSQC or HMBC experiments where necessary. Fluorine magnetic resonance spectra (^{19}F NMR) were recorded on a Bruker DPX-400 (162 MHz) instrument. Chemical shifts (δF) are quoted in ppm to the nearest 0.1 ppm and are referenced to CFCl3.

High resolution mass spectrometry (HRMS) was carried out on a Micromass LCT premier spectrometer using electron impact for all compounds.

Melting points were obtained on a Büchi B-545 melting point apparatus and are uncorrected.

Microwave reactions were carried out in a CEM Discover SP Microwave reactor in microwave vials equipped with clip-caps.

General Procedure 1 : Domino Heck-Suzuki Coupling using $Pd(dppf)Cl_2$

To an oven dried 10 mL microwave vial was added DMA (2 mL), 2M K_2CO_3 solution (0.15 mL), compound **1** (0.125 mmol) and boronic acid (0.225 mmol). The mixture was degassed with N_2. $Pd(dppf)Cl_2$ (0.063 mmol) was added and the mixture was heated at 150 °C in a CEM Discover SP microwave until TLC analysis indicated complete consumption of **1** (20-40 minutes depending upon boronic acid used). The mixture was filtered through Celite®, washing with EtOAc. Brine (10 mL) was added. The aqueous and organic layers were separated and the aqueous layer was extracted using EtOAc. The combined organic layers were dried ($MgSO_4$) and the solvent removed under reduced pressure. The resultant crude product material was purified by flash chromatography using the appropriate gradient of EtOAc and Petroleum Ether (40-60).

Compound Preparation and Characterisation

(*2R*,4aR*,9S*,9aR*,10R)-methyl 10-(benzo[b]thiophen-2-yl)-2,4a,9,9a-tetrahydro-1H-2,9-methanofluorene-3-carboxylate (4a)**

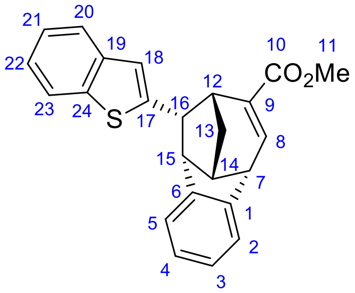

Prepared by general procedure 1 using benzo[b]thiophene-2-boronic acid. Title compound isolated as a clear film. R_f : 0.50; (SiO_2; 9 : 1, hexane : ethyl acetate); δ_H (500 MHz; $CDCl_3$): 7.55 (1H, dd, *J* = 8.0, 1.0 Hz, Ar-CH), 7.51-7.47 (1H, m, Ar-CH), 7.32 (1H, dd, *J* = 7.5, 1.0 Hz, Ar-CH), 7.19 (1H, ddd, *J* = 8.0, 7.0, 1.0 Hz, Ar-CH), 7.15-7.06 (3H, m, Ar-CH, C8-CH), 6.86 (1H, apparent td, *J* = 7.5, 1.0 Hz), 6.64 (1H, dd, *J* = 7.5, 1.0 Hz), 6.51 (1H, s, C18-CH), 4.00-3.90 (2H, m, C7-CH, C16-CH), 3.73 (1H, dd, *J* = 11.0, 6.5 Hz, C15-CH), 3.51 (1H, ddd, *J* = 5.5, 4.5, 1.0 Hz, C12-CH), 3.47 (3H, s, C11-CH_3), 3.43-3.39 (1H, m, C14-CH), 2.12 (1H, apparent dt, *J* = 11.5, 4.5 Hz, C13-CH_2), 1.87 (1H, d, *J* = 11.5 Hz, C13-CH_2); δ_c (125 MHz; $CDCl_3$): 167.0 (C10-C=O), 145.9 (Cq), 144.7 (Cq), 143.7 (Cq), 142.0 (C8-CH), 139.5 (Cq), 139.3 (Cq), 134.7 (Cq), 127.5 (Ar-CH), 127.2 (Ar-CH), 127.0 (Ar-CH), 124.3 (Ar-CH), 123.6 (Ar-CH), 123.2 (Ar-CH), 122.7 (Ar-CH), 122.1 (C18-CH) 121.9 (Ar-CH), 53.2 (C15-CH), 51.8 (C11-CH_3), 49.2 (C7-CH), 48.3 (C16-CH), 48.1 (C14-CH), 42.0 (C12-CH), 35.4 (C13-CH_2); ν_{max} (neat)/cm^{-1} 3061, 2946 (C-H), 1711 (C=O), 1435, 1248; HRMS : $(M+H)^+$ found , 395.1076 required 395.1076, Δ ppm −0.01.

(*2R*,4aR*,9S*,9aR*,10R)-methyl 10-phenyl-2,4a,9,9a-tetrahydro-1H-2,9-methanofluorene-3-carboxylate (4b)**

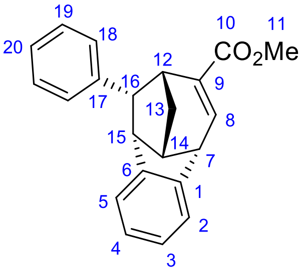

Prepared by general procedure 1 using phenylboronic acid. Title compound isolate as a white solid; R_f : 0.52 (SiO_2; 9 : 1, hexane : ethyl acetate); δ_H (400 MHz; $CDCl_3$): 7.29 (1H, dd, *J* = 7.5, 1.0 Hz), 7.13 (1H, apparent td, *J* = 7.5, 1.0 Hz, Ar-CH), 7.05-7.00 (1H, m, Ar-CH), 6.98-6.89 (4H, m, C8-CH, Ar-CH), 6.52-6.46 (3H, m, Ar-CH), 3.99 (1H, dd, *J* = 6.5, 4.5 Hz, C7-CH), 3.68 (1H, dd, *J* = 10.5, 6.5 Hz, C15-CH), 3.48 (1H, dd, *J* = 10.5, 5.5 Hz, C16-CH), 3.44-3.38 (4H, m, C14-CH, C11-CH_3), 3.36-3.32 (1H, m, C12-CH), 2.08 (1H, apparent dt, *J* = 11.5, 4.5 Hz, C13-CH_2), 1.83 (1H, d, *J* = 11.5 Hz, C13-CH_2); δ_c (100 MHz; $CDCl_3$): 166.9 (C10-C=O), 145.6 (Cq), 144.8 (Cq), 140.1 (C8-CH), 139.4 (Cq), 134.8 (Cq), 129.5 (Ar-CH), 127.5 (Ar-CH), 127.1 (Ar-CH), 126.9 (Ar-CH), 126.5 (Ar-CH), 126.1 (Ar-CH), 124.0 (Ar-CH), 52.5 (C15-CH), 52.0 (C16-CH), 51.5 (C11-CH_3), 49.2 (C7-CH), 48.1 (C14-CH), 42.3 (C12-CH), 35.4 (C13-CH_2); ν_{max} (neat)/cm^{-1} : 3054, 2948 (C-H), 1709 (C=O), 1634, 1436, 1246, 1070; HRMS : $(M+Na)^+$ found 339.1354, $C_{22}H_{20}O_2Na$ required 339.1356, Δ ppm −0.59; melting point : 107-109 °C.

(*2R*,4aR*,9S*,9aR*,10R)-methyl 10-(2-methoxyphenyl)-2,4a,9,9a-tetrahydro-1H-2,9-methanofluorene-3-carboxylate (4c)**

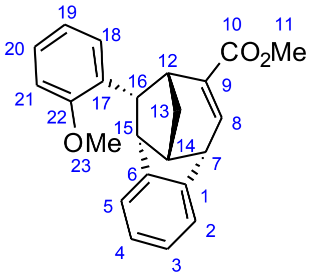

Prepared by general procedure 1 using 2-methoxyphenylboronic acid. Title compound isolated as a clear gum; R_f: 0.31 (SiO_2; 9 : 1, hexane : ethyl acetate); δ_H (500 MHz; $CDCl_3$): 7.26-7.23 (1H, m, C2/5-CH), 7.08 (1H, apparent td, *J* = 7.5, 1.0 Hz, C3/4-CH), 7.00 (1H, ddd (*J* = 8.0, 7.5, 1.5 Hz, C19-CH), 6.95 (1H, dd, *J* = 5.0, 1.0 Hz, C8-CH), 6.89 (1H, apparent td, *J* = 7.5, 1.0 Hz, C3/4-CH), 6.80 (1H, dd *J* = 8.0, 1.0 Hz, C18-CH), 6.52 (1H, dd, *J* = 7.5, 1.0 Hz, C2/5-CH), 6.37, (1H, apparent td, *J* = 7.5, 1.0 Hz (C20-CH), 5.94, (1H, dd, *J* = 7.5, 1.5 Hz, C21-CH), 4.13 (1H, dd, *J* = 11.0, 5.0 Hz, C16-CH), 3.96 (1H, dd, *J* = 6.5, 5.0 Hz, C7-CH), 3.87 3H, s, C23-CH_3), 3.73 (1H, dd, *J* = 11.0, 6.5 Hz, C15-CH); 3.49, (3H, s, C11-CH_3), 3.40 (1H, dddd, *J* = 6.5, 5.5, 4.5, 1.0 Hz, C14-CH), 3.32 (1H, m, C12-CH), 2.11 (1H, apparent dt, *J* = 11.5, 4.5 Hz, C13-CH_2), 1.8 (1H, d *J* = 11.5 Hz, C13-CH_2); δ_c (125 MHz; $CDCl_3$): 167.3 (C10-C=O), 157.4 (Cq) , 145.9 (Cq), 145.0 (Cq), 140.3 (C8-CH), 135.3

(Cq), 128.9 (C21-CH), 128.1 (Cq), 127.1 (C2/5-CH), 126.6 (C3/4-CH), 126.6 (C8-CH), 126.4 (C3/4-CH) , 123.9 (C2/5-CH), 119.3 (C20-CH), 109.7 (C18-CH) 55.7 (C11-CH_3), 51.5 (C23-CH_3), 51.3 (C15-CH), 49.2 (C7-CH), 47.8 (C14-CH), 41.9 (C16-CH), 40.6 (C12-CH), 35.9 (C13-CH_2) ν_{max} (neat)/cm^{-1} 3113, 2932, (C-H), 1717 (C=O), 1587, 1510, 1335; HRMS : $(M+Na)^+$ found 369.1459, $C_{23}H_{22}O_3Na$ required 369.1461, Δ ppm −0.64

(*2R*,4aR*,9S*,9aR*,10R)-methyl 10-(2-fluorophenyl)-2,4a,9,9a-tetrahydro-1H-2,9-methanofluorene-3-carboxylate (4d)**

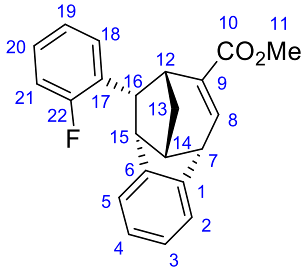

Prepared by general procedure 1 using 2-flurophenylboronic acid. Title compound isolated as a white solid; R_f **:** 0.43 (SiO_2; 9 : 1, hexane : ethyl acetate); δ_H (500 MHz; $CDCl_3$): 7.29-7.24 (1H, m), 7.12 (1H, apparent td, *J* = 7.5, 1.0 Hz, C21-CH), 7.01-6.90 (4H, m, C8-CH, Ar-CH) 6.57 (1H, d, *J* = 7.5 Hz), 6.54-6.48 (1H, m), 5.90 (1H, apparent td, *J* = 8.0, 1.5 Hz), 4.00-3.97 (2H, m, C7-CH, C16-CH), 3.72 (1H, dd, *J* = 11.0, 6.5 Hz, C15-CH), 3.46-3.40 (4H, m, C11-CH_3, C14-CH), 3.37-3.34 (1H, m C12-CH), 2.12 (1H, apparent td, *J* = 11.5, 4.5 Hz, C13-CH_2), 1.84 (1H, d, *J* = 11.5 Hz, C13-CH_2); δ_c (125 MHz; $CDCl_3$): 166.9 (C10-C=O), 161.0 (d, *J* = 243.0 Hz, C22-Cq), 145.0 (d, *J* = 40 Hz, C17-Cq), 140.1 (C8-CH), 134.7 (Cq), 129.7 (d, *J* = 3.5 Hz, Ar-CH), 127.3 (Ar-CH), 127.2 (d, *J* = 9.0 Hz, Ar-CH), 127.0 (Ar-CH), 126.6 (Ar-CH), 126.5 (Cq), 126.4 (Cq), 124.0 (Ar-CH), 122.5 (d, *J* = 3.5 Hz, Ar-CH), 114.3 (d, *J* = 24.0 Hz, Ar-CH), 51.6 (C15-CH), 51.5 (C11-CH_3), 49.2 (C7-CH), 47.9 (C14-CH), 40.9 (d, *J* = 4.0 Hz C16-CH), 40.9 (C12-CH), 35.6 (C13-CH_2); δ_F (125 MHz; $CDCl_3$): -119.3; ν_{max} (neat)/cm^{-1} : 2947 (C-H), 1710 (C=O), 1634, 1488, 1456, 1435, 1246, 1232; HRMS : $(M+H)^+$ found 357.1262, $C_{22}H_{19}O_2FNa$ required 357.1261, Δ ppm +0.31; melting point : 108-110 °C.

(*2R*,4aR*,9S*,9aR*,10R)-methyl 10-(4-fluorophenyl)-2,4a,9,9a-tetrahydro-1H-2,9-methanofluorene-3-carboxylate (4e)**

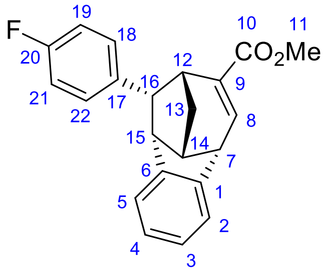

Prepared by general procedure 1 using 4-fluorophenylboronic acid. Title compound isolated as a yellow gum; R_f: 0.41 (SiO_2; 9 : 1, hexane : ethyl acetate); δ_H (400 MHz; $CDCl_3$): 7.31-7.24 (1H, m, Ar-CH), 7.14 (1H, apparent td, *J* = 7.5, 1.0 Hz, Ar-CH), 6.97-6.91 (2H, m, C8-CH, Ar-CH), 6.68-6.61 (2H, m, Ar-CH), 6.49 (1H, dd, *J* = 7.5, 1.0 Hz, Ar-CH), 6.45-6.38 (2H, m, Ar-CH, C18/22-CH), 3.99 (1H, dd, *J* = 6.5, 5.0 Hz, C7-CH), 3.65 (1H, dd, *J* = 11.0, 6.5 Hz, C15-CH), 3.48 (1H, dd, *J* = 11.0, 5.5 Hz, C16-CH), 3.45-3.36 (4H, m, C14-CH, C11-CH_3), 3.34-3.28 (1H, m, C12-CH), 2.07 (1H, apparent dt, *J* = 11.5, 4.5 Hz, C13-CH_2), 1.82 (1H, d, *J* = 11.5 Hz, C13-CH_2); δ_c - (100 MHz; $CDCl_3$): 166.8 (C10-C=O), 161.5 (d, *J* = 244.0 Hz, C20-Cq), 145.0 (d, *J* = 55.0 Hz ? Cq), 140.3 (CH), 135.1 (d, *J* = 3.0 Hz, Cq), 134.6 (Cq), 130.8 (CH), 130.8 (CH) 127.5 (CH), 127.0 (CH), 126.6 (CH), 124.1 (CH), 114.0 (CH), 113.8 (CH), 110.0 (Cq), 52.4 (C15-CH), 51.5 (C11-CH_3), 51.1 (C16-CH), 49.2 (C7-CH), 48.0 (C14-CH), 42.3 (C12-CH), 35.3 (C13-CH_2); δ_F (125 MHz; $CDCl_3$): -117.6; ν_{max} (neat)/cm^{-1} : 2917 (C-H), 1715 (C=O), 1509, 1248;

(*2R*,4aR*,9S*,9aR*,10R)-methyl 10-(benzo[d][1,3]dioxol-5-yl)-2,4a,9,9a-tetrahydro-1H-2,9-methanofluorene-3-carboxylate (4f)**

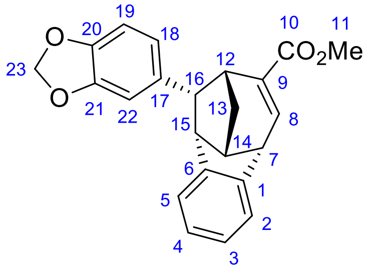

Prepared by general procedure 1 using 3,4-(methylenedioxy)phenylboronic acid. Title compound isolated as a clear gum; R_f : 0.31 (SiO_2; 9 : 1, hexane : ethyl acetate); δ_H (400 MHz; $CDCl_3$): 7.28 (1H, ddd, *J* = 7.5, 1.3, 0.5 Hz, C2/5-CH), 7.14 (1H, apparent td, *J* = 7.5, 1.0 Hz, C3/4-CH), 6.97 (1H, apparent td, *J* = 7.5, 1.0 Hz, C3/4-CH), 6.94 (1H, apparent dt, *J* = 4.7, 1.0 Hz, C8-CH), 6.57 (1H, dd, *J* = 7.5, 1.0

Hz, C2/5-C<u>H</u>), 6.45 (1H, d, *J* = 8.0 Hz, C19-C<u>H</u>), 6.06 (1H, dd, *J* = 8.0, 2.0 Hz, C18-C<u>H</u>), 5.89 (1H, d, *J* = 2.0 Hz, C22-C<u>H</u>), 5.78 (1H, d, *J* = 8.5 Hz, C23-C$\underline{H}_2$), 5.78 (1H, d, *J* = 8.5 Hz, C23-C$\underline{H}_2$) 3.97 (1H, dd, *J* = 6.3, 4.7 Hz, C7-C<u>H</u>), 3.63 (1H, dd, *J* = 10.5, 6.5 Hz, C15-C<u>H</u>), 3.48 (3H, s, C11-C$\underline{H}_3$), 3.44-3.36 (2H, m, C16-C<u>H</u>, C14-C<u>H</u>), 3.31 (1H, ddd, *J* = 5.5, 4.5, 1.0 Hz, C12-C<u>H</u>) 2.04 (1H, apparent dt, *J* = 11.5, 4.5, C13-C$\underline{H}_2$), 1.79 (1H, d, *J* = 11.5 Hz, C13-C$\underline{H}_2$); δ_C (100 MHz; $CDCl_3$): 167.0 (C10-<u>C</u>=O), 146.6 (<u>C</u>q), 145.8 (<u>C</u>q), 145.6 (<u>C</u>q), 144.9 (<u>C</u>q), 140.2 (C8-<u>C</u>H), 134.9 (<u>C</u>q), 133.5 (<u>C</u>q) 127.6 (C2/5-<u>C</u>H), 127.1 (C3/4-<u>C</u>H), 126.7 (C3/4-<u>C</u>H), 124.2 (C2/5-<u>C</u>H), 122.8 (C18-<u>C</u>H), 109.9 (C22-<u>C</u>H), 107.2 (C19-<u>C</u>H), 100.6 (C23-$\underline{C}H_2$), 52.6 (C15-<u>C</u>H), 51.8 (C16-<u>C</u>H), 51.7 (C11-$\underline{C}H_3$), 49.3 (C7-<u>C</u>H), 48.1 (C14-<u>C</u>H), 42.4 (C12-<u>C</u>H), 35.4 (C13-$\underline{C}H_2$); ν_{max} (neat)/cm^{-1} 2947 (C-H), 1708 (C=O), 1632, 1486, 1439, 1249; HRMS : $(M+Na)^+$ found 383.1253, $C_{23}H_{20}O_4Na$ required 383.1254, Δ ppm −0.31.

(*2R*,4aR*,9S*,9aR*,10R)-methyl 10-(4-phenoxyphenyl)-2,4a,9,9a-tetrahydro-1H-2,9-methanofluorene-3-carboxylate (4g)**

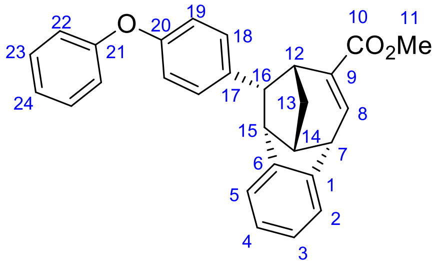

Prepared by general procedure 1 using (4-phenoxyphenyl)boronic acid. Title compound isolated as a clear film; R_f : 0.33 (SiO_2; 9 : 1, hexane : ethyl acetate); δ_H (500 MHz; $CDCl_3$): 7.29-7.24 (3H, m, Ar-C<u>H</u>), 7.12 (1H, apparent td, *J* = 7.5, 1.0 Hz, Ar-C<u>H</u>), 7.04-6.99 (1H, m, Ar-C<u>H</u>), 6.98-6.92 (2H, m, C8-C<u>H</u>, Ar-C<u>H</u>), 6.88 (2H, m, Ar-C<u>H</u>), 6.62 (2H, d, *J* = 8.5 Hz C18/19-C<u>H</u>), 6.55 (1H, dd, *J* = 7.5, 1.0 Hz, Ar-C<u>H</u>), 6.45 (2H, d, *J* = 8.5 Hz, C18/19-C<u>H</u>) 3.99 (1H, dd, *J* = 6.5, 4.5 Hz, C7-C<u>H</u>), 3.67 (1H, dd, *J* = 10.5, 6.5 Hz, C15-C<u>H</u>), 3.50 (1H, dd, *J* = 10.5, 5.5 Hz, C16-C<u>H</u>), 3.46 (3H, s, C11-$\underline{C}H_3$) 3.44-3.39 (1H, m, C14-C<u>H</u>), 3.34 (1H, apparent t, *J* = 5.0 Hz, C12-C<u>H</u>), 2.08 (1H, dt, *J* = 11.5, 4.5 Hz, C13-C$\underline{H}_2$), 1.83 (1H, d, *J* = 11.5 Hz, C13-C$\underline{H}_2$) δ_C (125 MHz; $CDCl_3$): 167.0 (C10-<u>C</u>=O), 157.9 (<u>C</u>q), 155.2 (<u>C</u>q), 145.6 (<u>C</u>q), 144.9 (<u>C</u>q), 140.4 (C8-<u>C</u>H), 134.8 (<u>C</u>q) 134.8 (<u>C</u>q), 130.8 (Ar-<u>C</u>H), 129.7 (Ar-<u>C</u>H) 127.7 (Ar-<u>C</u>H), 127.1 (Ar-<u>C</u>H), 126.7 (Ar-<u>C</u>H), 124.2 (Ar-<u>C</u>H), 122.8 (Ar-<u>C</u>H), 118.4 (Ar-<u>C</u>H), 118.1 (Ar-<u>C</u>H), 52.3 (C15-<u>C</u>H), 51.6 (C16-<u>C</u>H), 51.4 (C11-<u>C</u>H), 49.3 (C7-<u>C</u>H), 48.2 (C14-<u>C</u>H), 42.4 (C12-<u>C</u>H), 35.4 (C13-$\underline{C}H_2$); ν_{max} (neat)/cm^{-1} : 3021, 2945, 1709, 1588, 1505, 1489, 1273; HRMS : $(M+H)^+$ found 409.1798, $C_{28}H_{25}O_3$ required 409.1798, Δ ppm −0.13.

(*2R*,4aR*,9S*,9aR*,10R)-methyl 10-(3-cyanophenyl)-2,4a,9,9a-tetrahydro-1H-2,9-methanofluorene-3-carboxylate (4h)**

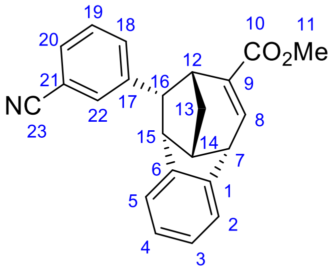

Prepared by general procedure 1 using 3-cyanophenylboronic acid. Title compound isolated as a clear gum; R_f : 0.46 (SiO_2; 9 : 1, hexane : ethyl acetate); δ_H (500 MHz; $CDCl_3$): 7.39-7.30 (2H, m, Ar-C<u>H</u>), 7.18 (1H, apparent t, *J* = 7.5 Hz, Ar-C<u>H</u>), 7.06-6.92 (3H, m, C8-C<u>H</u>, Ar-C<u>H</u>), 6.80 (1H, d, *J* = 1.5 Hz, Ar-C<u>H</u>), 6.62 (1H, dd, *J* = 8.0, 1.5 Hz, Ar-C<u>H</u>), 6.45 (1H, d, *J* = 7.5 Hz, Ar-C<u>H</u>), 4.02 (1H, dd, *J* = 6.5, 5.0 Hz, C7-C<u>H</u>), 3.70 (1H, dd, *J* = 10.5, 6.5 Hz, C15-C<u>H</u>), 3.50 (1H, dd, *J* = 10.5, 5.5 Hz, C16-C<u>H</u>), 3.45 (4H, m, C11-C<u>H</u>$_3$, C14-C<u>H</u>) 3.35 (1H, apparent t, *J* = 5.0 Hz, C12-C<u>H</u>), 2.10 (1H, apparent dt, *J* = 11.5 Hz, C13-C<u>H</u>$_2$), 1.86 (1H, d, *J* = 11.5 Hz, C13-C<u>H</u>$_2$); δ_c (125 MHz; $CDCl_3$): 166.6 (C10-<u>C</u>=O), 144.6 (<u>C</u>q), 141.0 (<u>C</u>q), 140.6 (C8-<u>C</u>H), 134.1 (<u>C</u>q), 134.1 (<u>C</u>q) 133.9 (Ar-<u>C</u>H), 133.2 (Ar-<u>C</u>H), 130.0 (Ar-<u>C</u>H), 127.9 (Ar-<u>C</u>H), 127.6 (Ar-<u>C</u>H), 127.3 (Ar-<u>C</u>H), 126.8 (Ar-<u>C</u>H), 124.5 (Ar-<u>C</u>H), 119.0 (C23-<u>C</u>≡N), 111.2 (C21-<u>C</u>q), 52.4 (C15-<u>C</u>H), 51.6 (C11-<u>C</u>H$_3$), 51.3 (C16-<u>C</u>H), 49.1 (C7-<u>C</u>H), 48.0 (C14-<u>C</u>H), 42.2 (C12-<u>C</u>H), 35.3 (C13-<u>C</u>H$_2$) ν_{max} (neat)/cm^{-1} : 2948 (C-H), 2228 (C≡N), 1708 (C=O), 1633, 1475, 1435, 1273, 1247 ; HRMS : $(M+Na)^+$ found 364.1298, $C_{23}H_{19}O_2NNa$ required 364.1308, Δ ppm −2.72.

(*2R*,4aR*,9S*,9aR*,10R)-methyl 10-(2,6-dimethylphenyl)-2,4a,9,9a-tetrahydro-1H-2,9-methanofluorene-3-carboxylate (4i)**

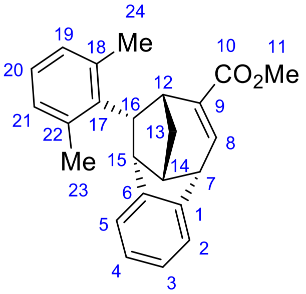

Prepared by general procedure 1 using 2,6-dimethylphenylboronic acid. Title compound isolated as a white solid; R_f : 0.52 (SiO_2; 9 : 1, hexane : ethyl acetate); δ_H (400 MHz; $CDCl_3$) 7.23 (1H, dd, *J* = 7.5, 1.0 Hz, Ar-C<u>H</u>), 7.12 (1H, dd, *J* = 7.5, 1.0

Hz, Ar-CH), 7.02 (1H, dd, *J* = 7.0, 1.0 Hz, Ar-CH), 6.96-6.88 (3H, m C8-CH, Ar-CH), 6.69 (1H, dd, *J* = 7.5, 1.0 Hz, Ar-CH), 4.09-4.03 (1H, m, C7-CH), 4.00 (1H, dd, *J* = 10.5, 4.5 Hz, C16-CH) 3.67-3.58 (4H, m, C11-CH_3, C15-CH), 3.48-3.42 (1H, m, C15-CH), 3.40 (1H, apparent td, *J* 4.5, 1.0 Hz, C14-CH), 2.49 (3H, s, C23/C24-CH_3), 2.10 (1H, apparent dt, *J* = 11.0, 4.0 Hz, C13-CH_2), 1.83 (1H, d, *J* = 11.0 Hz, C13-CH_2), 1.03 (3H, s, C23/C24-CH_3); δ_c (100 MHz; $CDCl_3$): 167.3 (C10-C=O), 145.5 (Cq), 144.2 (Cq), 139.5 (Cq), 139.2 (C8-CH), 138.0 (Cq), 137.8 (Cq), 136.4 (Cq), 130.2 (Ar-CH), 128.0 (Ar-CH), 126.9 (Ar-CH), 126.7 (Ar-CH), 126.4 (Ar-CH), 125.7 (Ar-CH), 124.0 (Ar-CH), 51.7 (C11-CH_3), 50.7 (C16-CH), 50.2 (C7-CH), 49.3 (C15-CH), 45.7 (C14-CH), 40.5 (C12-CH), 39.4 (C13-CH), 22.6 (C23/24-CH_3), 21.9 (C23/24-CH_3); ν_{max} (neat)/cm^{-1} : 3021, 2949 (C-H), 1709 (C=O), 1631, 1475, 1455, 1485, 1266, 1236; HRMS : $(M+Na)^+$ found , 367.1660, $C_{24}H_{24}O_2Na$ required 367.1669, Δ ppm −2.29; melting point : 175-177 °C.

(*2R*,4aR*,9S*,9aR*,10R)-methyl 10-(thiophen-3-yl)-2,4a,9,9a-tetrahydro-1H-2,9-methanofluorene-3-carboxylate (4j)**

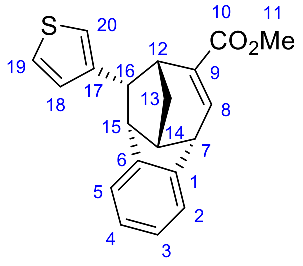

Prepared by general procedure 1 using 3-thienylboronic acid. Title compound isolated as pale yellow solid; R_f **:** 0.48 (SiO_2; 9 : 1, hexane : ethyl acetate); δ_H (500 MHz; $CDCl_3$): 7.28-7.26 (1H, m, C2/C5-C<u>H</u>), 7.12 (1H, apparent td, *J* = 7.5, 1.0 Hz, C2/5-C<u>H</u>), 6.98-6.91 (2H, m, C8-C<u>H</u>, C3/4-C<u>H</u>), 6.88 (1H, dd, *J* = 5.0, 3.0 Hz, C19-C<u>H</u>), 6.62-6.60 (1H, m, C2/5-C<u>H</u>), 6.24 (1H, dd, *J* = 3.0, 1.5 Hz, C20-C<u>H</u>), 6.16 (1H, dd, *J* = 5.0, 1.5 Hz, C18-C<u>H</u>), 3.96-3.93 (1H, m, C7-C<u>H</u>), 3.71 (1H, dd, *J* = 11.0, 5.5 Hz, C16-C<u>H</u>), 3.62 (1H, dd, *J* = 11.0, 6.5 Hz, C15-C<u>H</u>), 3.44 (3H, s, C11-C<u>H</u>$_3$), 3.40-3.35 (1H, m, C14-C<u>H</u>), 3.31 (1H, apparent td, *J* = 4.5, 2.5 Hz, C12-C<u>H</u>), 2.04 (1H, apparent dt, *J* = 11.5, 4.5 Hz, C13-C<u>H</u>$_2$), 1.82 (1H, d, *J* = 11.5 Hz, C13-C<u>H</u>$_2$); δ_c (125 MHz; $CDCl_3$): 166.8 (C10-<u>C</u>=O), 145.6 (<u>C</u>q), 145.3 (<u>C</u>q), 140.5 (C8-<u>C</u>H), 140.0 (Cq), 135.0 (<u>C</u>q), 128.8 (C18-<u>C</u>H), 127.4 (C2/5-<u>C</u>H), 126.9 C2/5-<u>C</u>H), 126.6 (C3/4-<u>C</u>H), 124.0 (C2/C5-<u>C</u>H), 123.0 (C19-<u>C</u>H), 121.5 (C20-<u>C</u>H), 52.3 (C15-<u>C</u>H), 51.5 (C11-<u>C</u>H$_3$), 49.1 (C7-<u>C</u>H), 48.0 (C14-<u>C</u>H), 47.1 (C16-<u>C</u>H), 42.0 (C12-<u>C</u>H), 34.8 (C13-<u>C</u>H$_2$); ν_{max} (neat)/cm^{-1} : 2945 (C-H), 1707 (C=O), 1634, 1475, 1435, 1434, 1246, ; HRMS : $(M+Na)^+$ found 345.0917, $C_{20}H_{18}O_2NaS$ required 345.0920, Δ ppm −0.68; melting point: 109-112 °C.

(*2R*,4aR*,9S*,9aR*,10R)-methyl 10-(pyridin-4-yl)-2,4a,9,9a-tetrahydro-1H-2,9-methanofluorene-3-carboxylate (4k)**

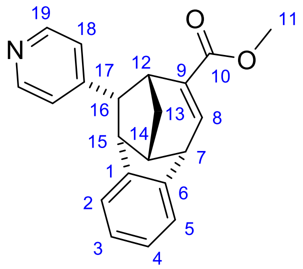

Prepared by general procedure 1 using 3-pyridinylboronic acid. Title compound isolated as an amorphous white solid; $\mathbf{R_f}$: 0.38 (SiO_2; 7:3 EtOAc/Pet. Ether (40-60)); $\boldsymbol{\delta_H}$ (400 MHz; $CDCl_3$): 8.18 – 8.14 (2H, m, C19-C<u>H</u>, C19'-C<u>H</u>), 7.29 (1H, d, *J* = 7.5 Hz, Ar-C<u>H</u>), 7.14 (1H, app td, *J* = 7.5, 1.0 Hz, Ar-C<u>H</u>), 6.97 (1H, d, *J* = 4.8, C8-H),

6.98 - 6.95 (1H, m, Ar-C<u>H</u>), 6.93 (1H, app td, J = 7.5, 1.0 Hz, Ar-C<u>H</u>), 6.47 (1H, d, J = 7.5 Hz, Ar-C<u>H</u>), 6.39 - 6.34 (2H, m, Ar-C<u>H</u>), 3.99 (1H, dd, J = 6.1, 4.8 Hz, C7-C<u>H</u>), 3.69 (1H, dd, J = 10.6, 6.5 Hz, C15-C<u>H</u>), 3.45 - 3.38 (2H, m, C14-C<u>H</u>, C16-C<u>H</u>),3.41 (3H, s, C11-C<u>H</u>$_3$), 3.37 - 3.33 (1H, m, C12-C<u>H</u>), 2.08 (1H, dt, J = 11.2, 4.3 Hz, C13-C<u>H</u>$_2$), 1.84 (1H, d, J = 11.2 Hz, C13-C<u>H</u>$_2$) ppm; **δ$_C$** (125 MHz; $CDCl_3$): 166.6 (C=O), 148.64 (C_q), 148.62 (C19-<u>C</u>H), 144.7 (<u>C</u>$_q$), 144.6 (<u>C</u>$_q$), 140.6 (C8-<u>C</u>H), 134.2 (<u>C</u>$_q$), 127.4 (Ar-<u>C</u>H), 126.9 (Ar-<u>C</u>H), 124.8 (C18-<u>C</u>H), 124.3 (Ar-<u>C</u>H), 52.4 (C15-<u>C</u>H), 51.6 (C11-<u>C</u>H$_3$), 51.2 (C14/C16-<u>C</u>H), 49.1 (C7-<u>C</u>H), 48.2 (C14/C16-<u>C</u>H), 42.0 (C12-<u>C</u>H), 35.4 (C13-<u>C</u>H$_2$) ppm; **v$_{max}$** (neat)/cm^{-1}: 2948 (C-H), 1709 (C=O), 1633, 1596, 1248, 1069; **HRMS**: $(M+H)^+$ found 318.1478, $C_{21}H_{20}O_2N$ required 318.1489, Δ ppm -3.2.

(*2R*,4aR*,9S*,9aR*,10R)-methyl 10-(1-phenylvinyl)-2,4a,9,9a-tetrahydro-1H-2,9-methanofluorene-3-carboxylate (4l)**

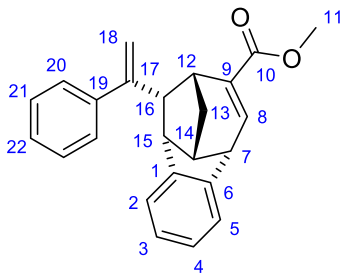

Prepared by general procedure 1 using 1-phenylvinyl boronic acid. Title compound isolated as an amorphous white solid. **R$_f$** : 0.45 (SiO_2; 1:9 EtOAc /Pet. Ether (40-60)); **δ$_H$** (500 MHz; $CDCl_3$): 7.35 – 7.30 (3H, m, Ar-C<u>H</u>), 7.29 - 7.22 (3H, m, Ar-C<u>H</u>), 7.11 (1H, app td, J = 7.5, 0.9 Hz, Ar-C<u>H</u>), 7.00 (1H, app td, J = 7.5, 0.9 Hz, Ar-C<u>H</u>), 6.93 (1H, d, J = 4.9 Hz, C8-C<u>H</u>), 6.64 (1H, d, J = 7.3 Hz, Ar-C<u>H</u>), 4.89 (1H, s, C18-C<u>H</u>$_2$), 3.98 (1H, s, C18-C<u>H</u>$_2$), 3.93 – 3.88 (1H, m, C7-C<u>H</u>), 3.69 - 3.63 (1H, m, C16-C<u>H</u>), 3.65 (3H, s, C11-C<u>H</u>$_3$), 3.49 - 3.45 (1H, m, C15-C<u>H</u>), 3.45 - 3.41 (1 H, m, C12-C<u>H</u>), 3.37 – 3.31 (1H, m, C14-C<u>H</u>), 2.00 (1H, dt, J = 11.6, 4.3 Hz, C13-C<u>H</u>$_{2a}$), 1.79 (1H, d, J = 11.6 Hz, C13-C<u>H</u>$_{2b}$) ppm; **δ$_C$** (125 MHz; $CDCl_3$): 167.3 (C=O), 145.6 (<u>C</u>$_q$), 145.5 (<u>C</u>$_q$), 145.3 (<u>C</u>$_q$), 145.1 (<u>C</u>$_q$), 140.3 (C8-<u>C</u>H), 134.8 (<u>C</u>$_q$), 128.2 (Ar-<u>C</u>H), 127.7 (Ar-<u>C</u>H), 126.9 (Ar-<u>C</u>H), 126.8 (Ar-<u>C</u>H), 126.4 (Ar-<u>C</u>H), 126.3 (Ar-<u>C</u>H), 123.8 (Ar-<u>C</u>H), 115.4 (C18-<u>C</u>H$_2$), 51.7 (C16-<u>C</u>H), 51.6 (C11-<u>C</u>H$_3$), 49.9 (C12-<u>C</u>H), 49.1 (C7-<u>C</u>H), 47.9 (C14-<u>C</u>H), 40.3 (C15-<u>C</u>H), 35.4 (C13-<u>C</u>H$_2$) ppm; **v$_{max}$** (neat)/cm^{-1}: 2947 (C-H), 1706 (C=O), 1633, 1435, 1244, 1069; **HRMS**: $(M+H)^+$ found 343.1694, $C_{24}H_{23}O_2$ required 343.1693, Δ ppm 0.5.

(*2R*,4aR*,9S*,9aS*,10R)-methyl 10-((E)-styryl)-2,4a,9,9a-tetrahydro-1H-2,9-methanofluorene-3-carboxylate (4m)**

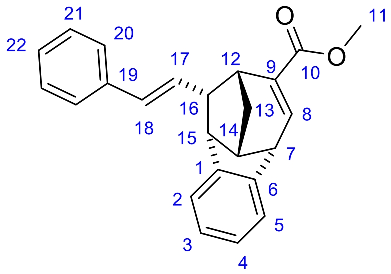

Prepared by general procedure 1 using *trans*-2-phenylvinylboronic acid. Title compound isolated as an amorphous white solid; R_f : 0.38 (SiO_2; 1:9 EtOAc/Pet. (40-60)); δ_H (400 MHz; $CDCl_3$): 7.26 – 7.22 (2H, m, Ar-CH), 7.20 - 7.13 (2H, m, Ar-CH), 7.14 – 7.09 (2H, m, Ar-CH), 7.07 – 7.00 (3H, m, Ar-CH), 6.92 (1H, d, *J* = 4.6 Hz, C8-CH), 6.45 (1H, d, *J* = 15.7 Hz, C18-CH), 5.22 (1H, dd, *J* = 15.7, 10.3 Hz, C17-CH), 3.89 (1H, dd, *J* = 5.9, 4.6 Hz, C7-CH), 3.58 – 3.53 (1H, dd , *J* = 10.3, 6.6 Hz, C15-CH), 3.65 (3H, s, C11-CH_3), 3.33 – 3.26 (1H, m, C14-CH), 3.21 (1 H, t, *J* = 4.9 Hz, C12-CH), 3.07 (1H, app td, , *J* = 10.3, 4.9 Hz, C16-CH), 2.04 – 1.96 (1H, m, C13-CH_{2a}), 1.77 (1H, d, *J* = 11.5 Hz, C13-CH_{2b}) ppm; δ_C (100 MHz; $CDCl_3$): 167.1 (C=O), 145.5 (C_q), 144.6 (C_q), 141.3 (C8-CH), 134.3 (C_q), 134.8 (C_q), 130.7 (C17-CH), 130.4 (Ar-CH), 128.3 (Ar-CH), 127.1 (Ar-CH), 126.8 (Ar-CH), 126.73 (Ar-CH), 126.71 (Ar-CH), 126.2 (Ar-CH), 124.0 (Ar-CH), 52.7 (C15-CH), 51.7 (C11-CH_3), 49.2 (C15-CH), 49.1 (C7-CH), 47.8 (C14-CH), 41.2 (C12-CH), 35.0 (C13-CH_2) ppm; ν_{max} (neat)/cm^{-1}: 2947 (C-H), 2331, 1708 (C=O), 1633, 1245, 1067; HRMS: $(M+H)^+$ found 343.1708, $C_{24}H_{23}O_2$ required 343.1698, Δ ppm 2.9.

(*2R*,4aR*,9S*,9aR)-methyl 10-phenyl-2,4a,9,9a-tetrahydro-1H-2,9-methanofluorene-3-carboxylate (4b) *via* tandem Heck-Stille**

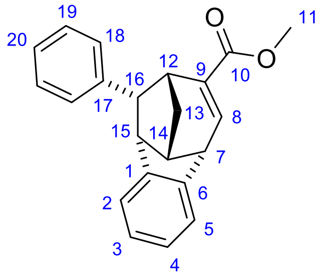

To an oven dried round bottomed flask was added compound **1** (40 mg, 0.125 mmol), tri-(2-furyl)phosphine (3 mg, 0.013 mmol), LiCl (10.6 mg, 0.250 mmol) and CH_3CN (2 mL). The mixture was degassed with N_2. $Pd_2(dba)_3$ (5.7 mg, 0.006 mmol) and tributylphenyl tin (62 µL, 0.188 mmol) were added and the mixture was heated at

80 °C for 18 h. Additional $Pd_2(dba)_3$ (5.7 mg, 0.006 mmol), tri-(2-furyl)phosphine (3 mg, 0.013 mmol) and LiCl (10.6 mg, 0.250 mmol) were added and the mixture was heated at 80 °C for a further 20 h. The solvent was removed *in vacuo*. KF solution (10 mL) was added and stirred for 30 mins. The organics were extracted using EtOAc (15 mL x 3). The combined organic layers were washed with KF soln, dried ($MgSO_4$) and the solvent removed under reduced pressure. The sample was purified by flash column chromatography using 10% w/v KF in 60 (230–240) mesh silica, eluting with a 0 -5 % EtOAc / Pet. Ether (60-40) gradient to yield a colourless oil (17.1 mg, 43% yield).

(*2R*,4aR*,9R*,9aS)-methyl 10-2,4a,9,9a-tetrahydro-1H-2,9-methanofluorene-3-carboxylate (9)**

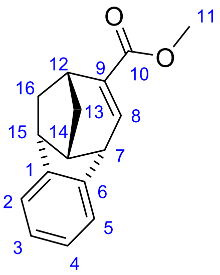

To a solution of compound **1** (50 mg, 0.16 mmol) in toluene (5 mL) was added AIBN (6.4 mg, 0.04 mmol) and $SnBu_3H$ (110 µL, 0.39 mmol). The solution was refluxed at 120 °C for 15 h. Additional $SnBu_3H$ (42 µL, 0.23 mmol) and AIBN (6.4 mg, 0.04 mmol) were added and the mixture was refluxed for a further 38 h. The solvent was concentrated *in vacuo*. KF solution (15 mL) was added and stirred for 30 mins. The organics were extracted using EtOAc (3 x 15 mL). The combined organic layers were washed with KF solution, dried over $MgSO_4$ and the solvent removed *in vacuo*. The sample was purified by flash column chromatography using 10% w/v KF in 60 (230–240) mesh silica. The column was first flushed with 3 column volumes of 100% Pet. Ether (40-60), then the product was eluted with a 3 -6 % EtOAc / Pet. Ether gradient to yield the product as a colourless oil (14.2 mg, 38% yield).

R_f : 0.41 (SiO_2; 1:9 EtOAc/Pet. Ether (40-60)); **δ_H** (500 MHz; $CDCl_3$): 7.23 - 7.18 (2H, m, Ar-CH), 7.14 (1H, app td, *J* = 7.3, 1.5 Hz, Ar-CH), 7.10 (1H, app td, *J* = 7.3, 1.5 Hz, Ar-CH), 6.83 (1H, d, *J* = 4.7 Hz, C8-CH), 3.81 (1H, dd, *J* = 6.1, 4.7 Hz, C7-CH), 3.65 (3H, s, C11-CH), 3.43 (1H, dd, *J* = 8.9, 2.3 Hz, C15-CH), 3.26 - 3.21 (1 H, m, C14-CH), 3.16 - 3.12 (1H, m, C12-CH), 2.17 (1H, ddd, *J* = 13.1, 11.3, 6.1 Hz, C16-CH_{2a}), 1.87 (1H, dt, *J* = 11.4, 4.5 Hz, C13-CH_{2b}), 1.73 (1H, dd, *J* = 11.4, 0.8 Hz, C13-CH_{2a}), 1.45 (1H, dt, *J* = 13.1, 2.3 Hz, C16-CH_{2b}) ppm; **δ_C** (125 MHz; $CDCl_3$): 167.1 (C=O), 148.7 (C_q), 146.2 (C_q), 141.1 (C8-CH), 137.7 (C_q), 127.2 (Ar-CH), 126.4 (Ar-

CH), 125.3 (Ar-CH), 124.1 (Ar-CH), 51.5 (C11-CH_3), 48.6 (C7-CH), 48.0 (C15-CH), 47.3 (C14-CH), 37.4 (C13-CH_2), 36.6 (C12-CH), 35.4 (C16 –CH_2) ppm; $\mathbf{v}_{max}$ (neat)/cm^{-1}: 2947 (C-H), 1707 (C=O), 1633, 1248, 1069; **HRMS**: $(M+H)^+$ found 241.1228, $C_{16}H_{16}O_2$ required 241.1229, Δ ppm -0.4.

NMR Spectra of Novel Compounds:

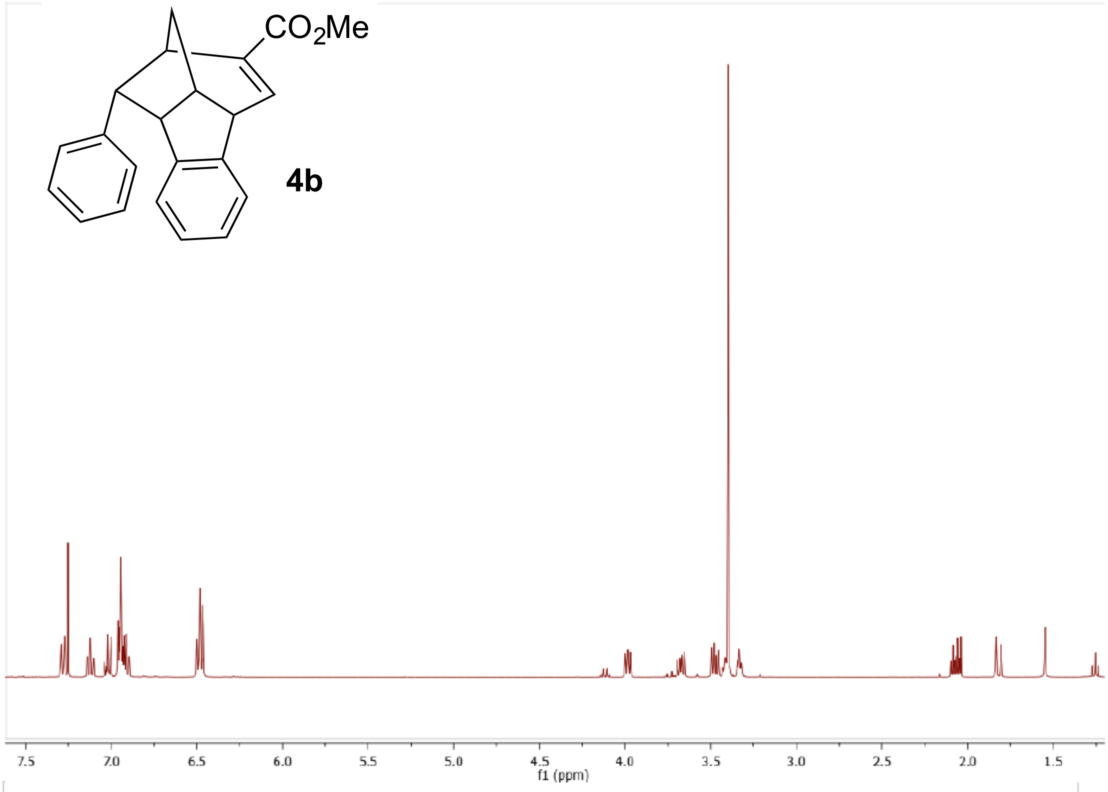

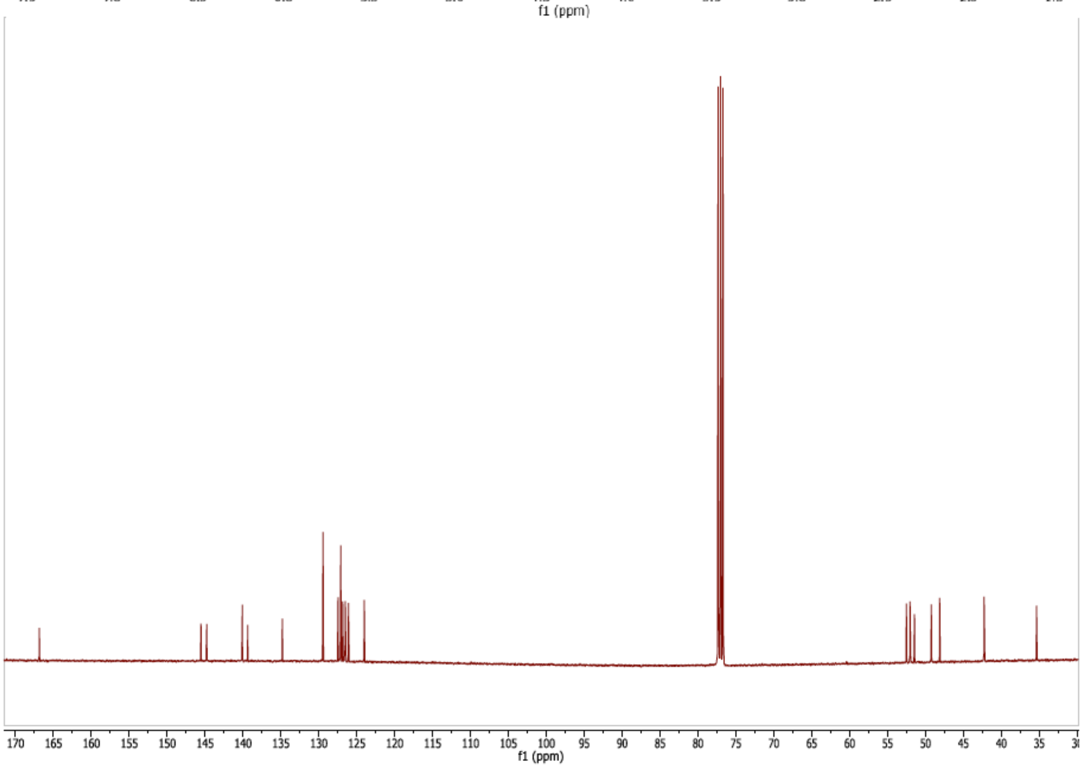

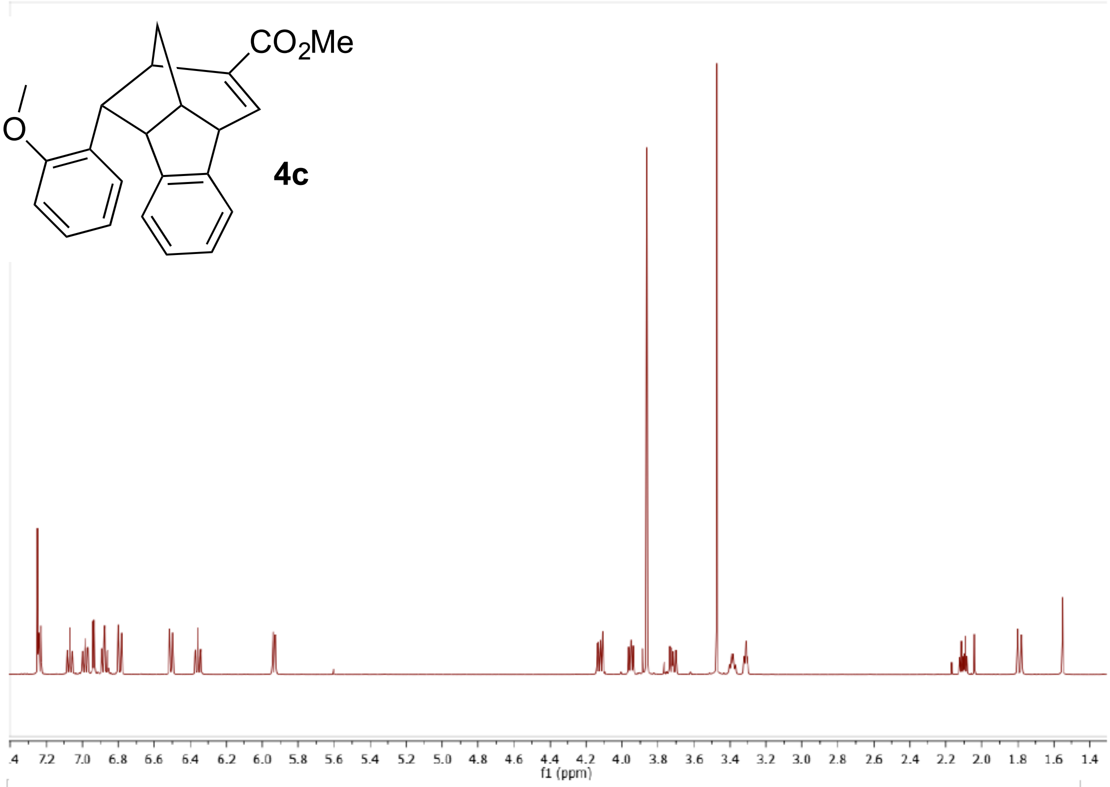
CO2Me
O
4c
f1 (ppm)

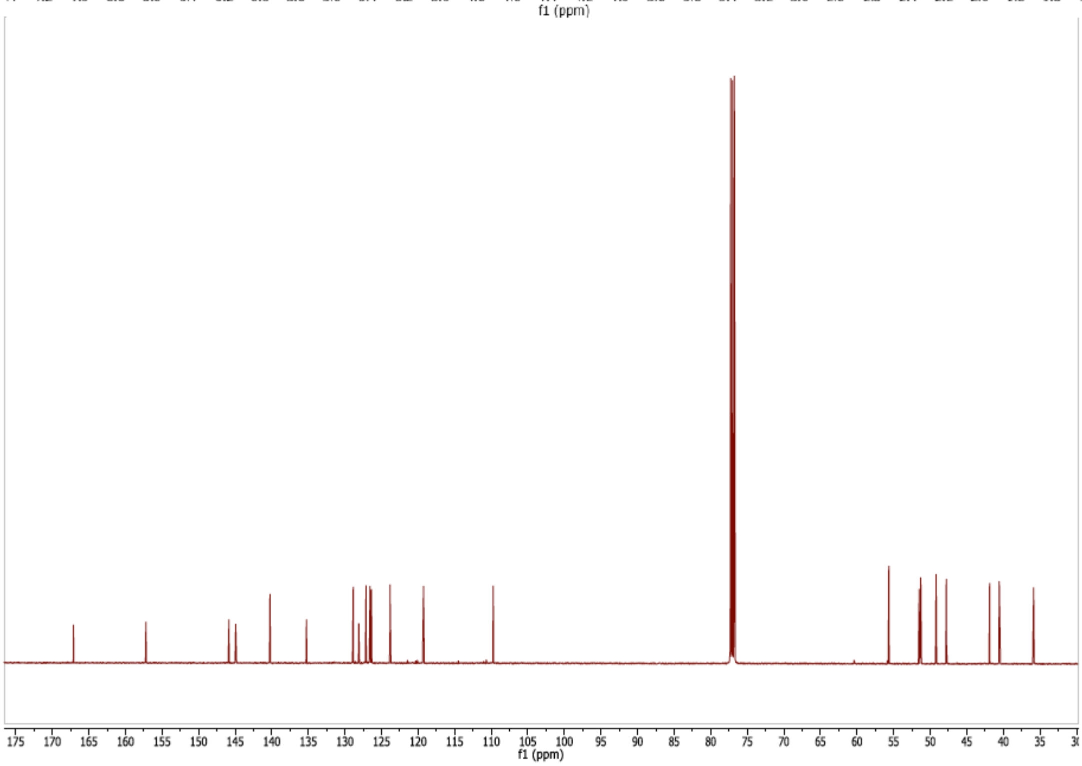
f1 (ppm)

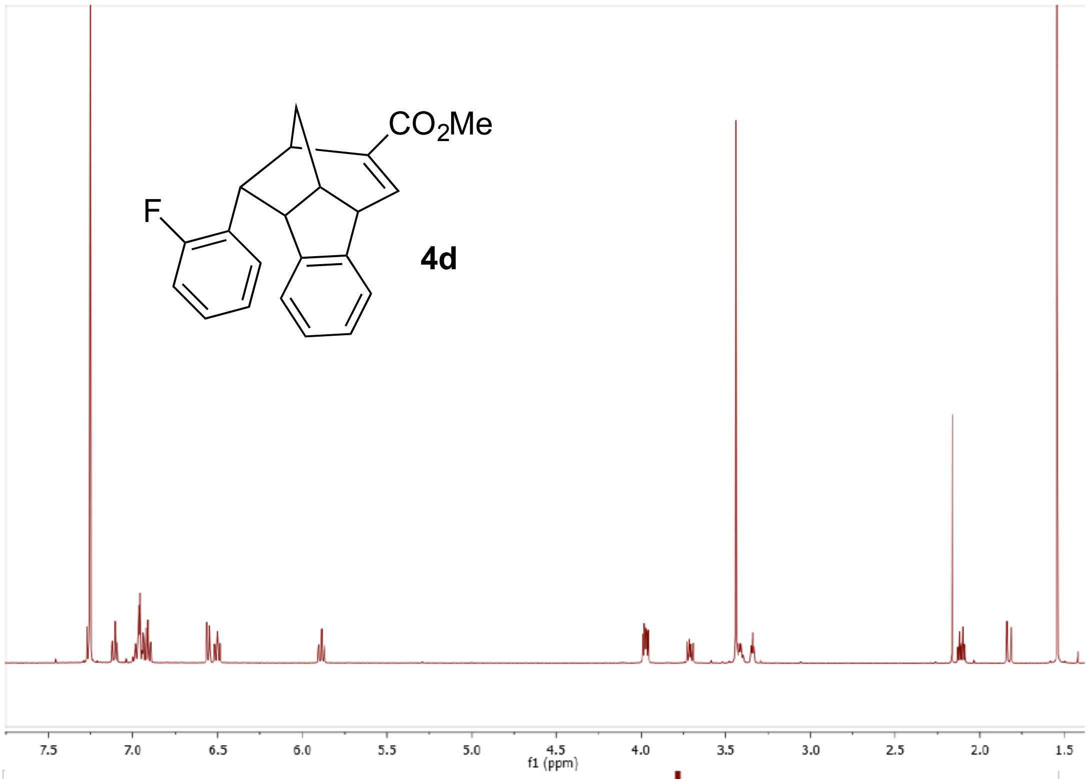
CO2Me
F
4d
7.5 7.0 6.5 6.0 5.5 5.0 4.5 4.0 3.5 3.0 2.5 2.0 1.5
f1 (ppm)

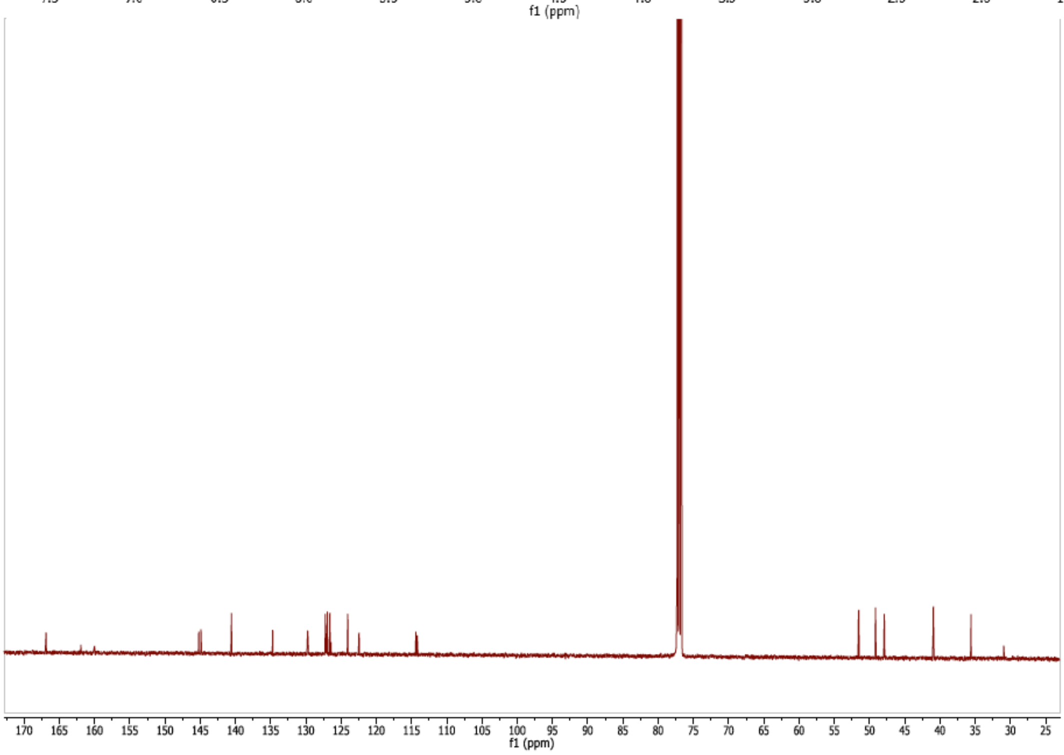
170 165 160 155 150 145 140 135 130 125 120 115 110 105 100 95 90 85 80 75 70 65 60 55 50 45 40 35 30 25
f1 (ppm)

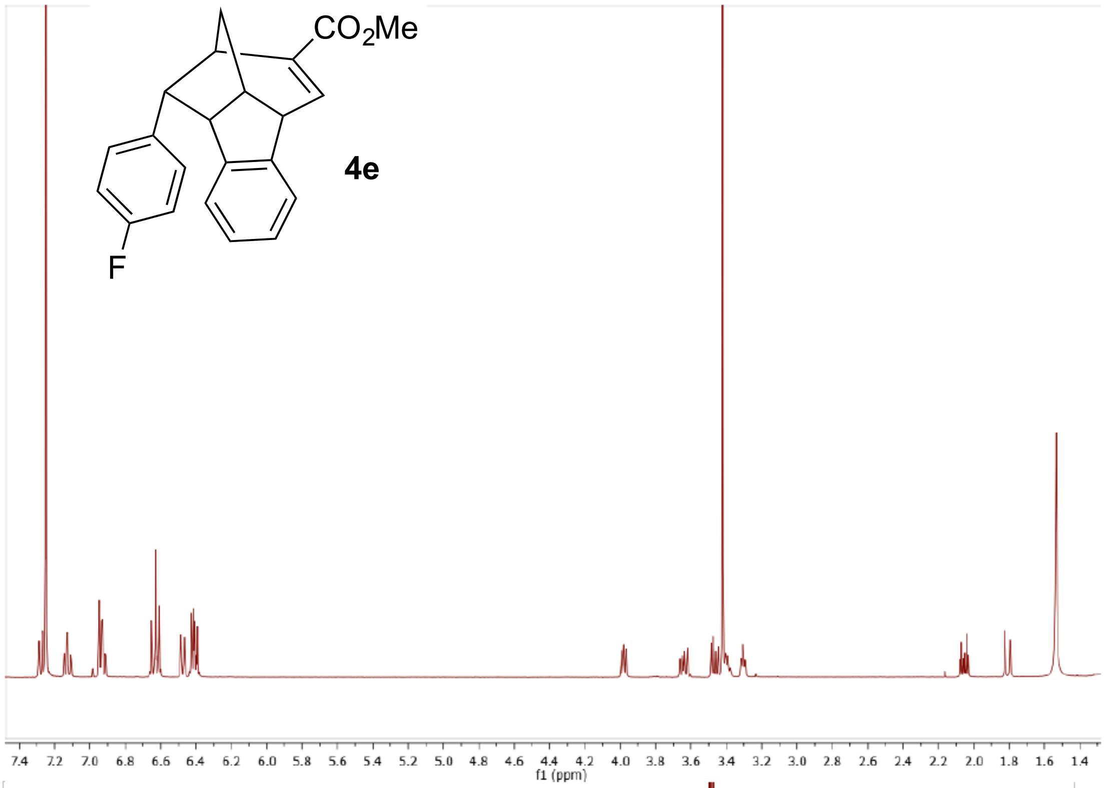
CO2Me
F
4e
7.4 7.2 7.0 6.8 6.6 6.4 6.2 6.0 5.8 5.6 5.4 5.2 5.0 4.8 4.6 4.4 4.2 4.0 3.8 3.6 3.4 3.2 3.0 2.8 2.6 2.4 2.2 2.0 1.8 1.6 1.4
f1 (ppm)

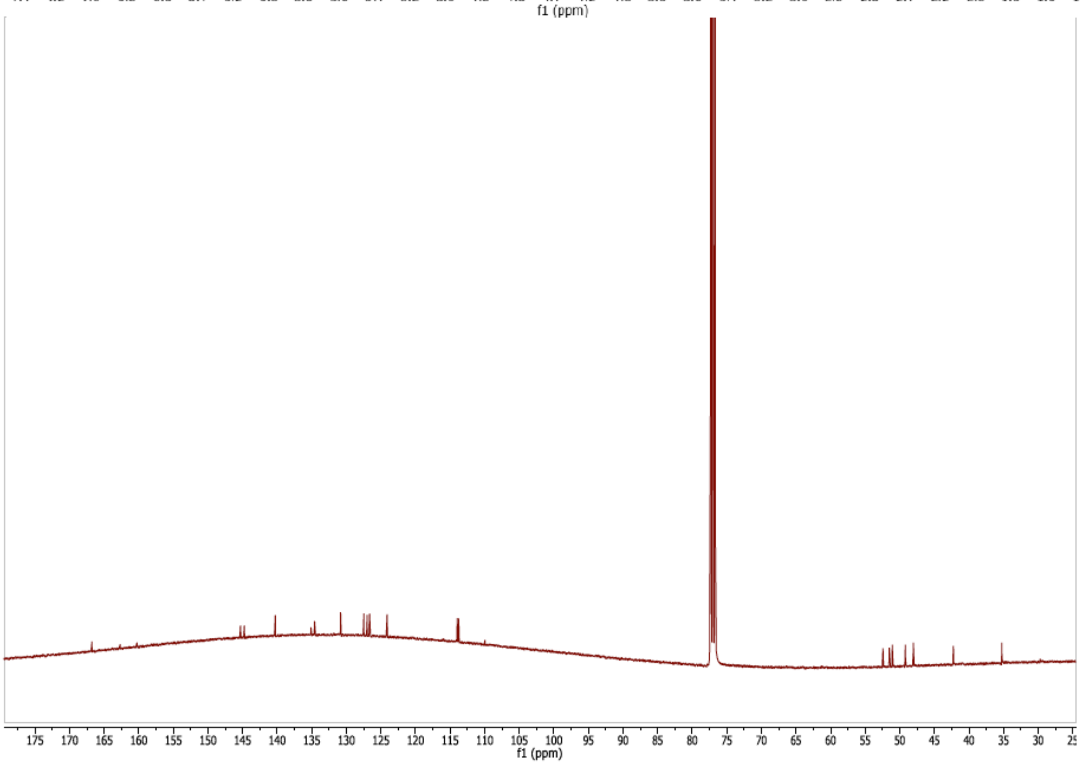
175 170 165 160 155 150 145 140 135 130 125 120 115 110 105 100 95 90 85 80 75 70 65 60 55 50 45 40 35 30 25
f1 (ppm)

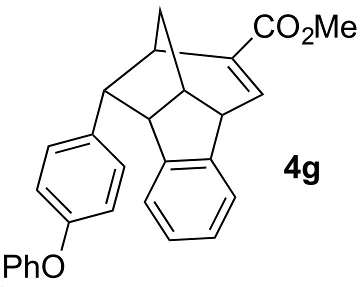
CO2Me
PhO

4g

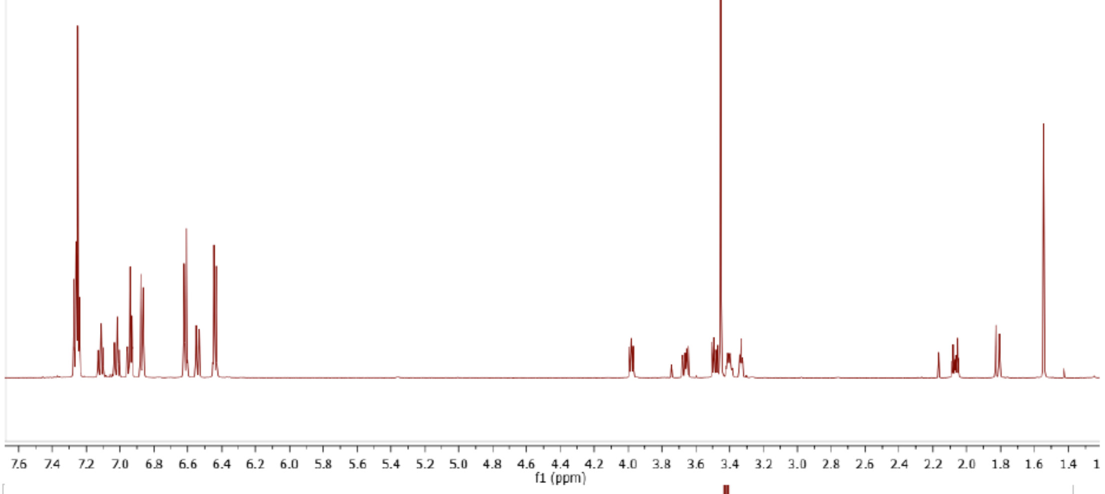
7.6 7.4 7.2 7.0 6.8 6.6 6.4 6.2 6.0 5.8 5.6 5.4 5.2 5.0 4.8 4.6 4.4 4.2 4.0 3.8 3.6 3.4 3.2 3.0 2.8 2.6 2.4 2.2 2.0 1.8 1.6 1.4 1
f1 (ppm)

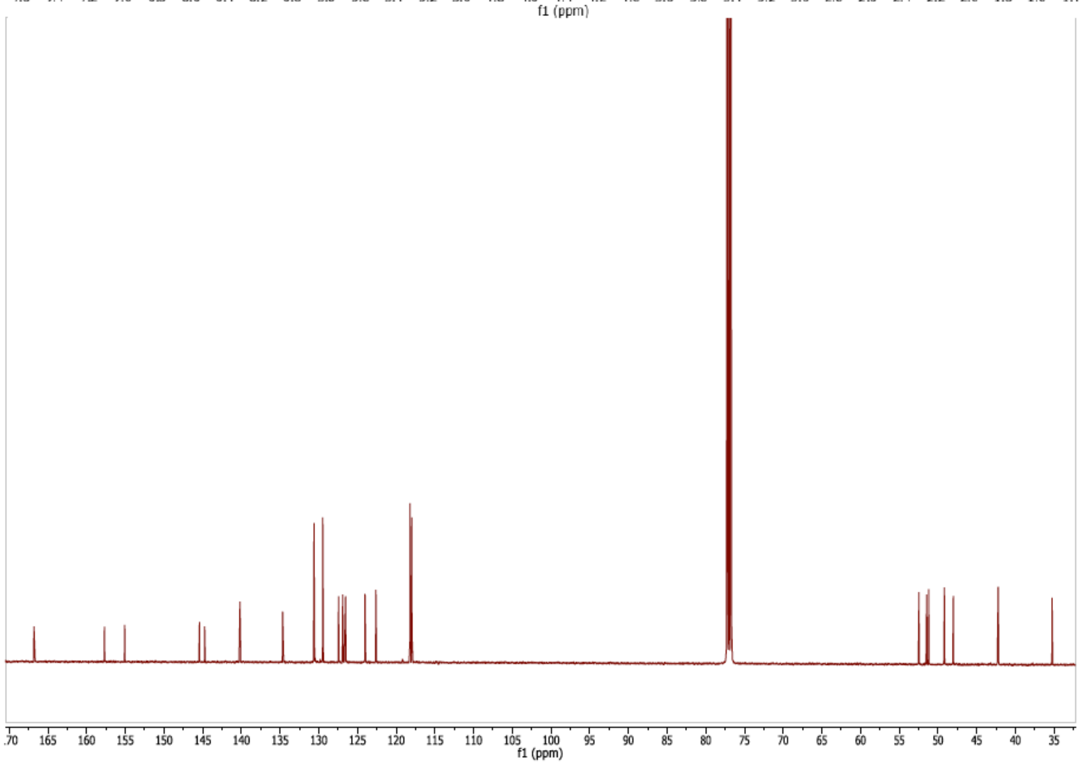
70 165 160 155 150 145 140 135 130 125 120 115 110 105 100 95 90 85 80 75 70 65 60 55 50 45 40 35
f1 (ppm)

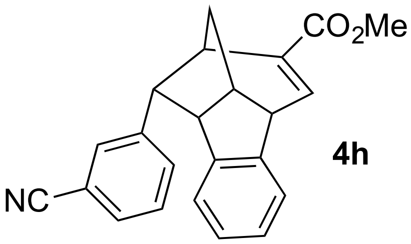
CO2Me
NC

4h

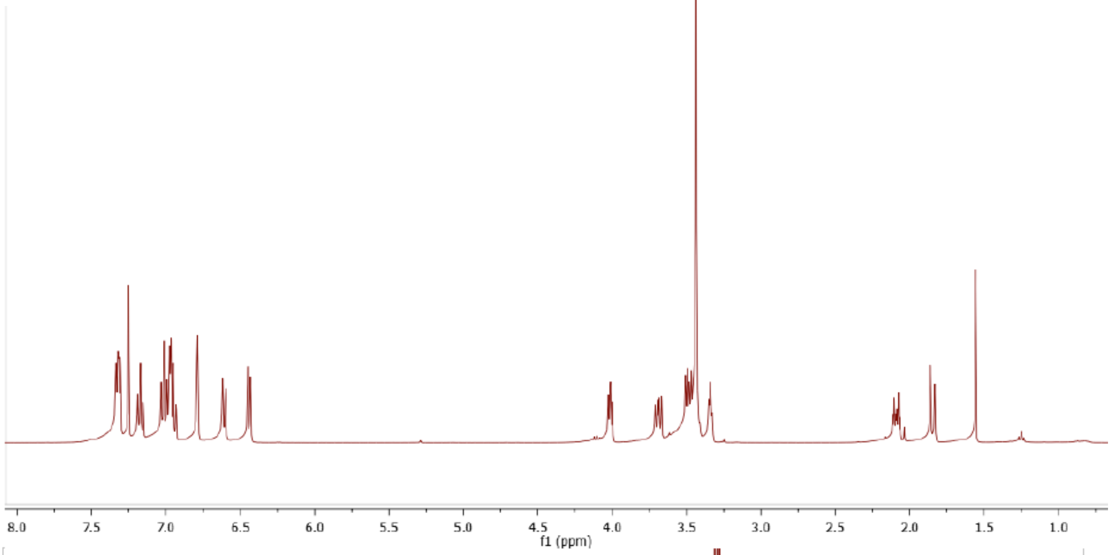
8.0
7.5
7.0
6.5
6.0
5.5
5.0
4.5
4.0
3.5
3.0
2.5
2.0
1.5
1.0
f1 (ppm)

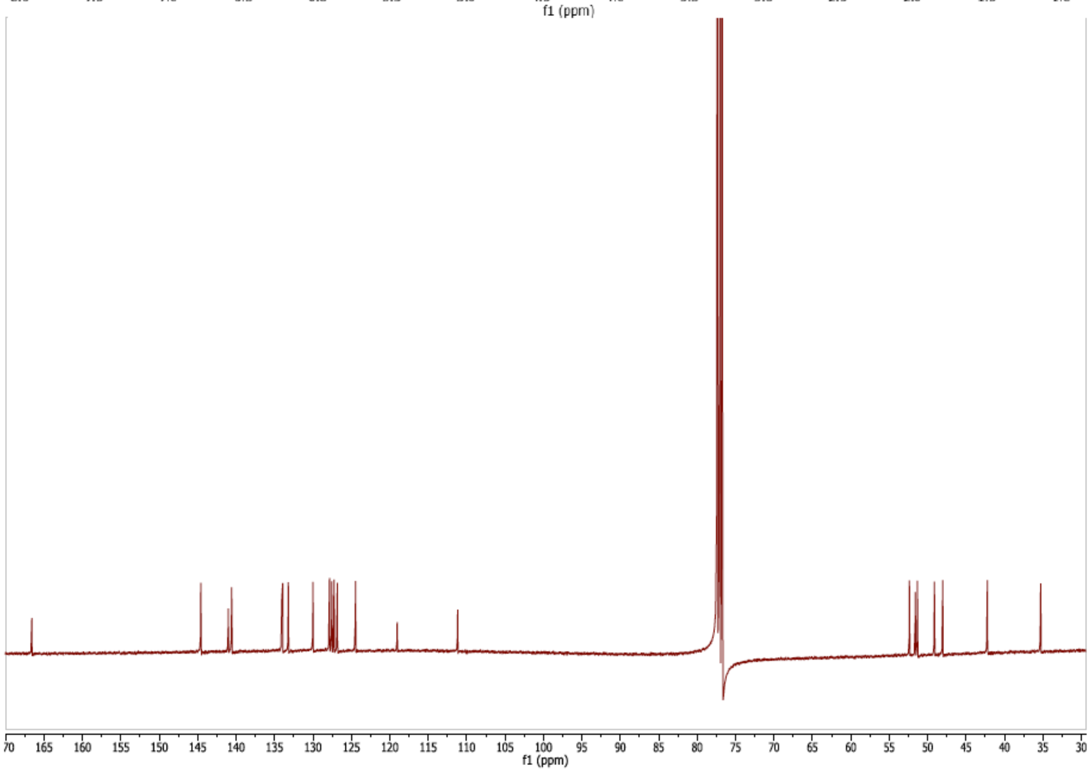
70
165
160
155
150
145
140
135
130
125
120
115
110
105
100
95
90
85
80
75
70
65
60
55
50
45
40
35
30
f1 (ppm)

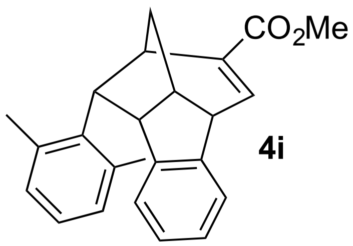
CO2Me

4i

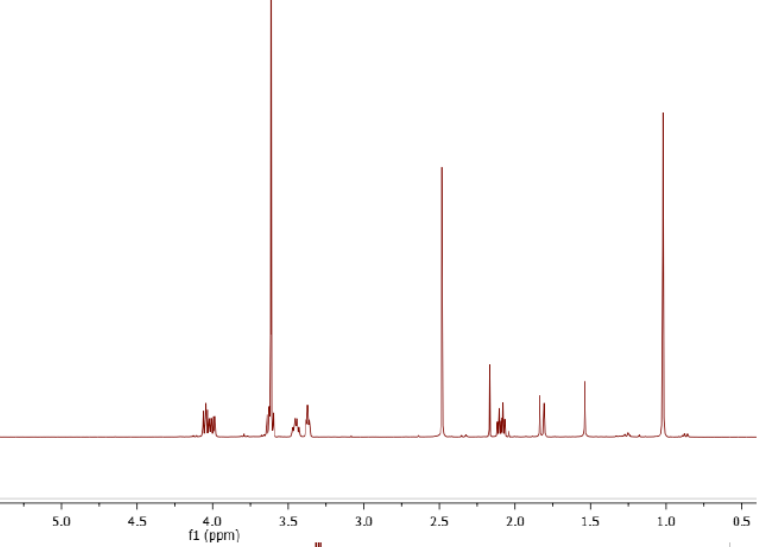
7.5
7.0
6.5
6.0
5.5
5.0
4.5
4.0
3.5
3.0
2.5
2.0
1.5
1.0
0.5
f1 (ppm)

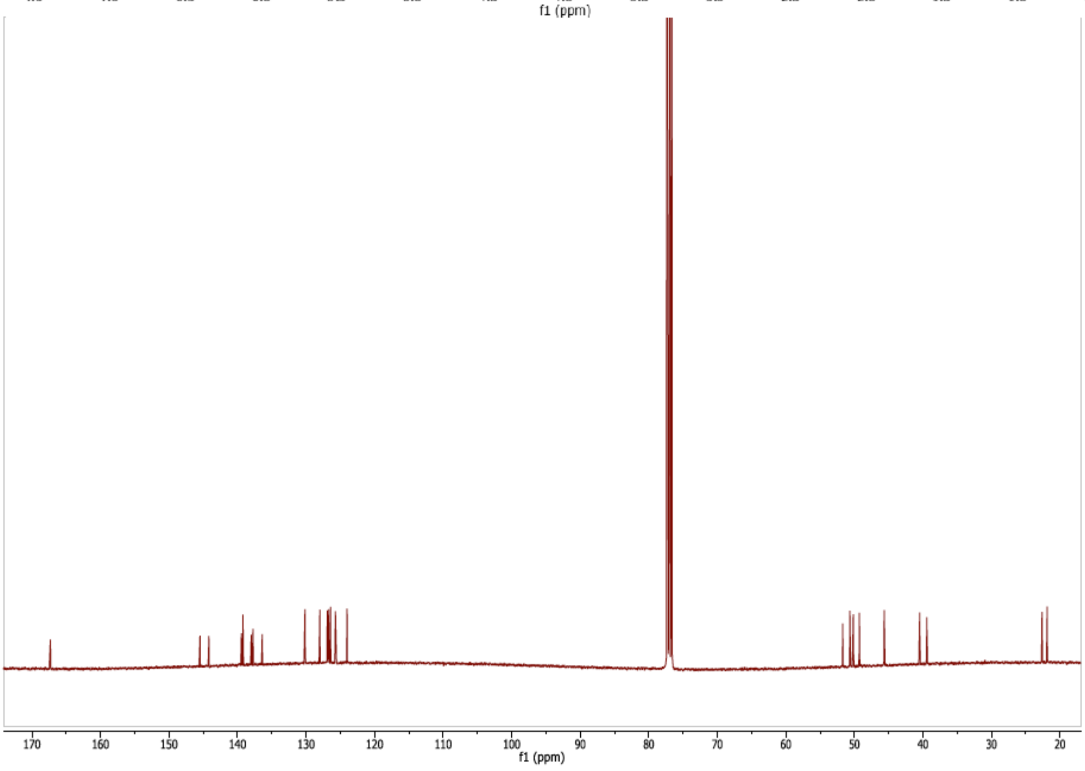
170
160
150
140
130
120
110
100
90
80
70
60
50
40
30
20
f1 (ppm)

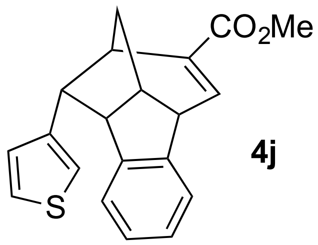
CO2Me
S

4j

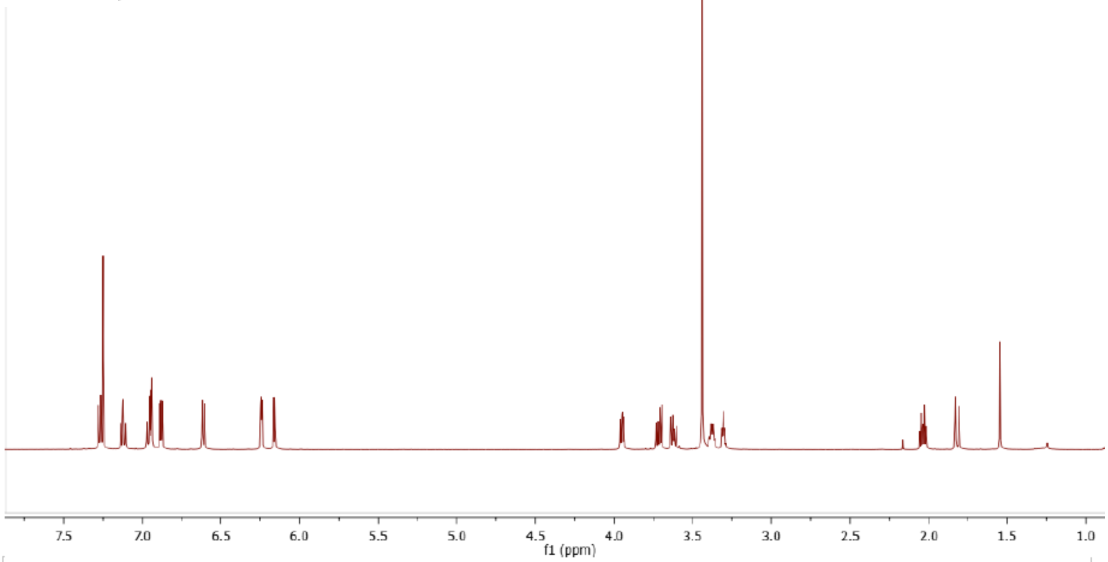
7.5
7.0
6.5
6.0
5.5
5.0
4.5
4.0
3.5
3.0
2.5
2.0
1.5
1.0
f1 (ppm)

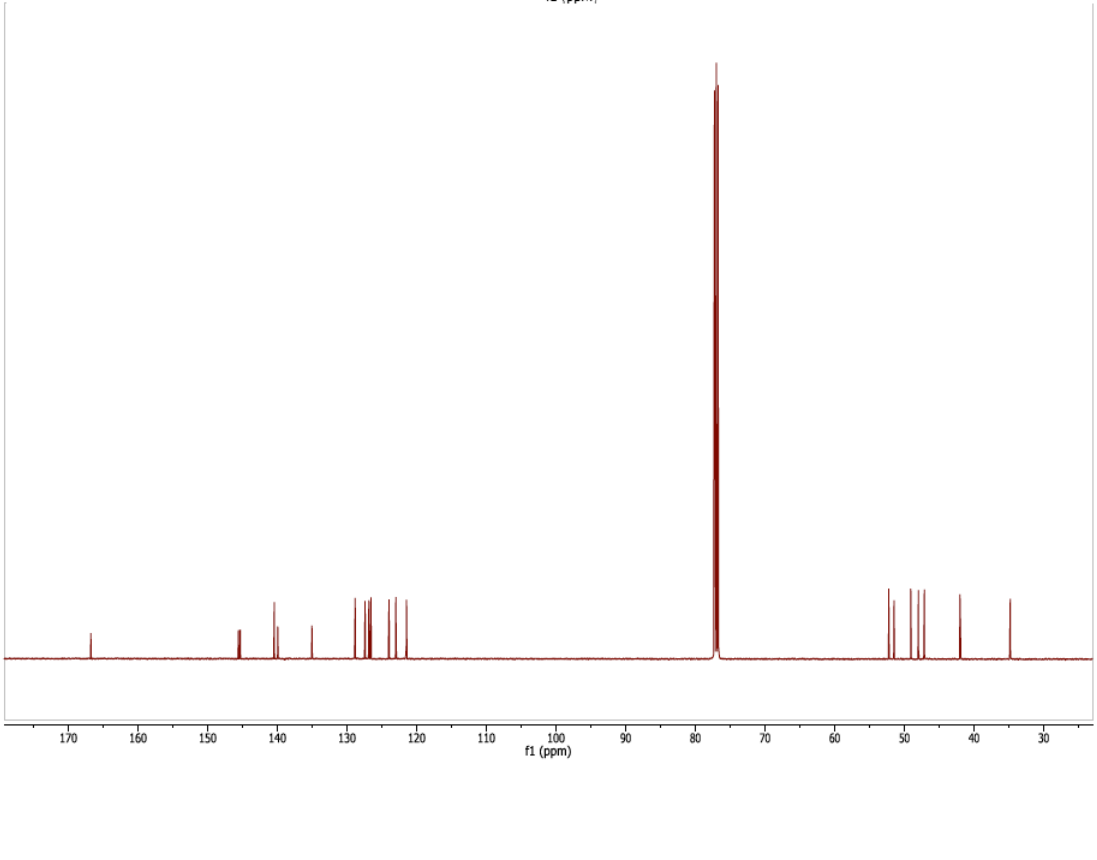
170
160
150
140
130
120
110
100
90
80
70
60
50
40
30
f1 (ppm)

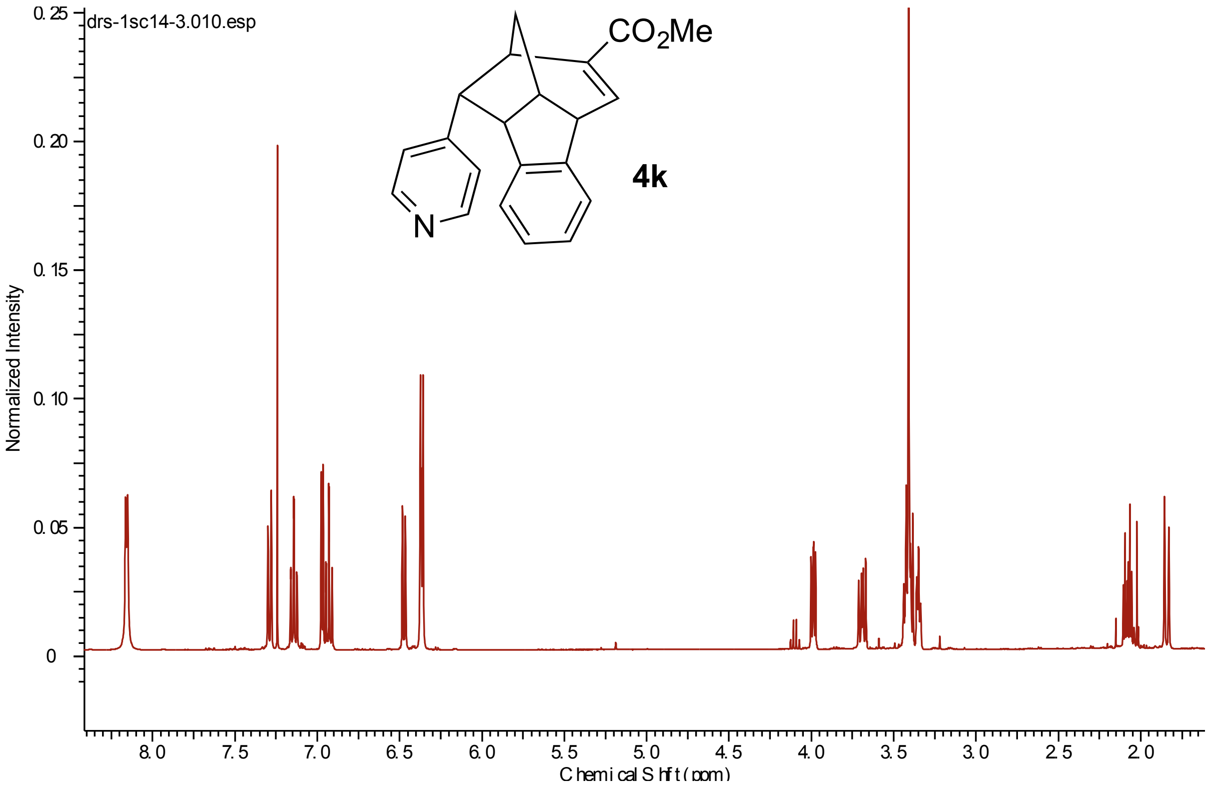
drs-1sc14-3.010.esp
CO2Me
N
4k
Normalized Intensity
Chemical Shift (ppm)

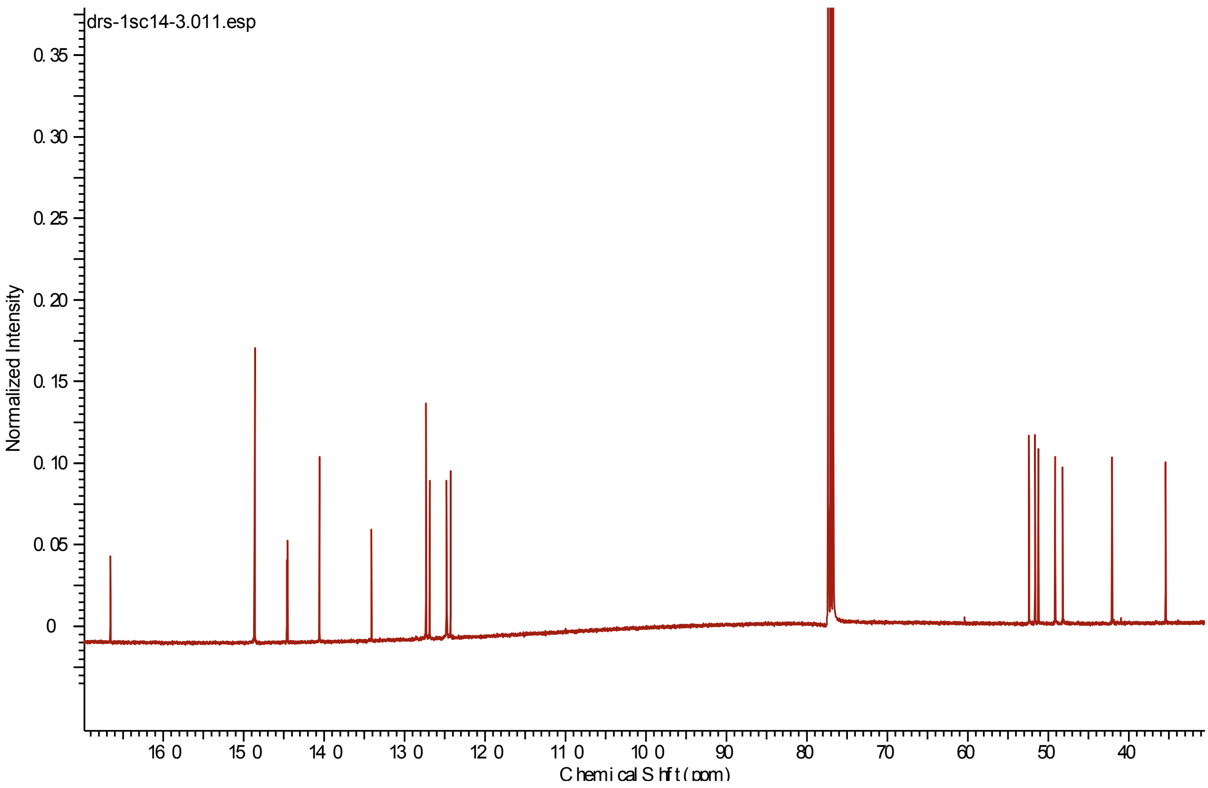
drs-1sc14-3.011.esp
Normalized Intensity
Chemical Shift (ppm)

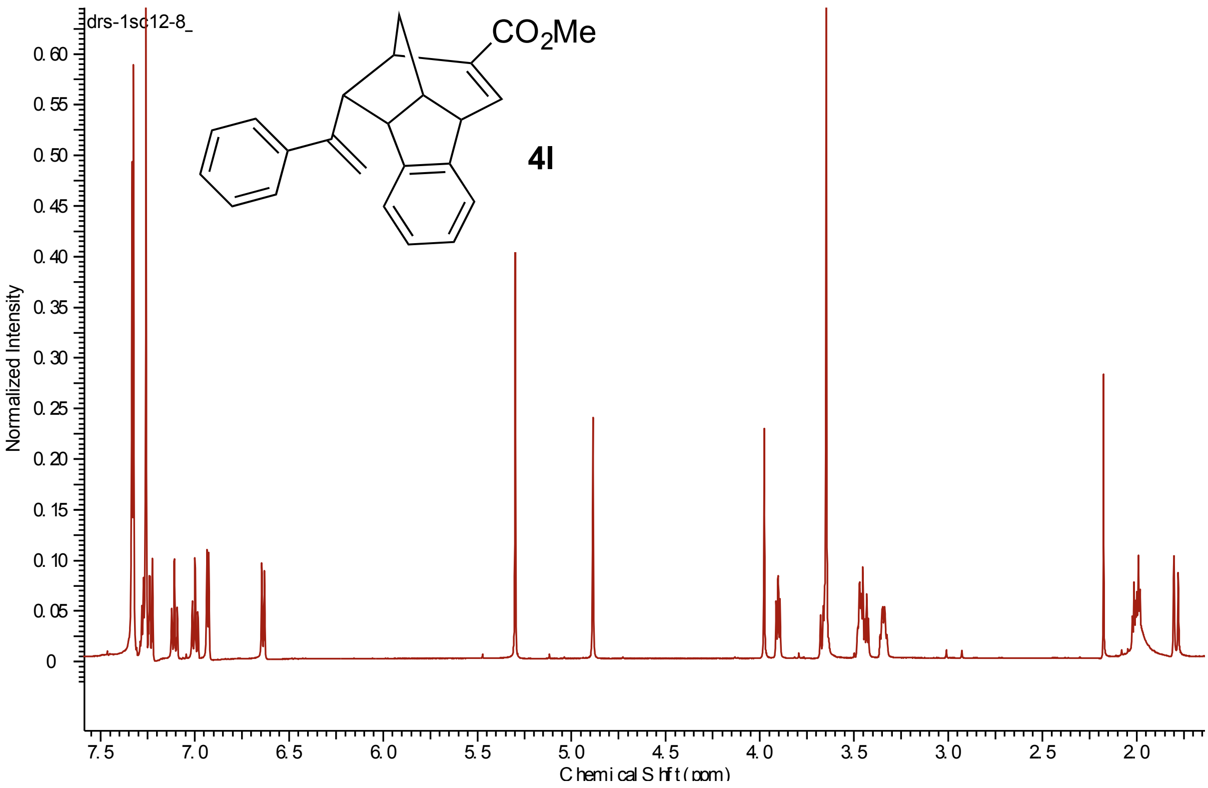
drs-1sc12-8_
CO_2Me
4l
Normalized Intensity
0.60
0.55
0.50
0.45
0.40
0.35
0.30
0.25
0.20
0.15
0.10
0.05
0
7.5 7.0 6.5 6.0 5.5 5.0 4.5 4.0 3.5 3.0 2.5 2.0
Chemical Shift (ppm)

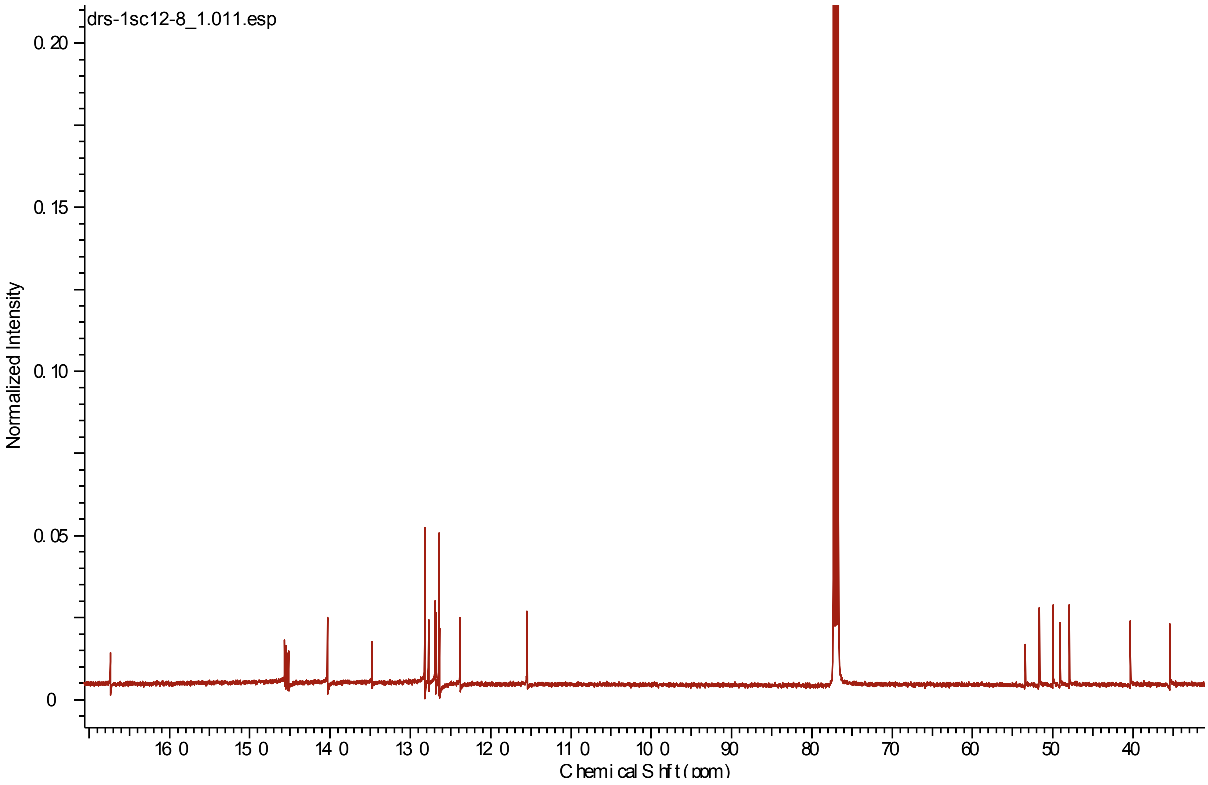
drs-1sc12-8_1.011.esp
Normalized Intensity
0.20
0.15
0.10
0.05
0
160 150 140 130 120 110 100 90 80 70 60 50 40
Chemical Shift (ppm)

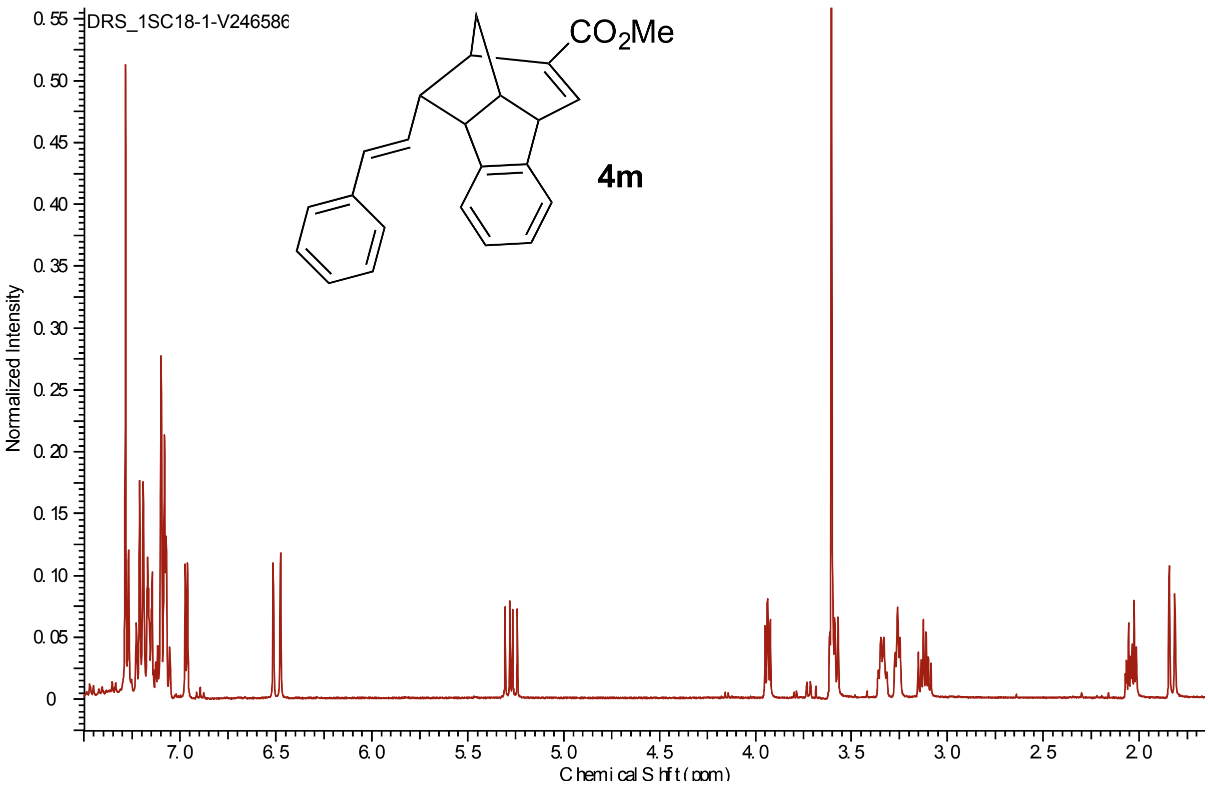
DRS_1SC18-1-V246586
CO2Me
4m
Normalized Intensity
Chemical Shift (ppm)

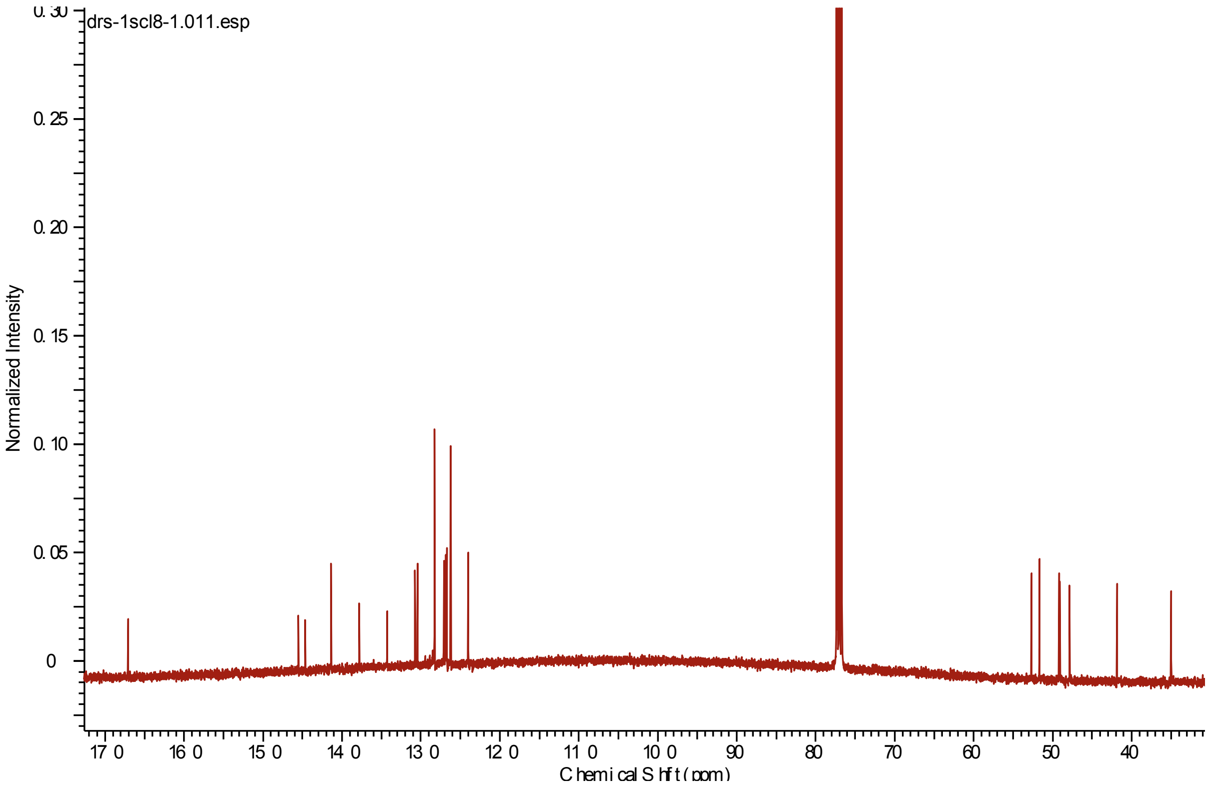
drs-1scl8-1.011.esp
Normalized Intensity
Chemical Shift (ppm)

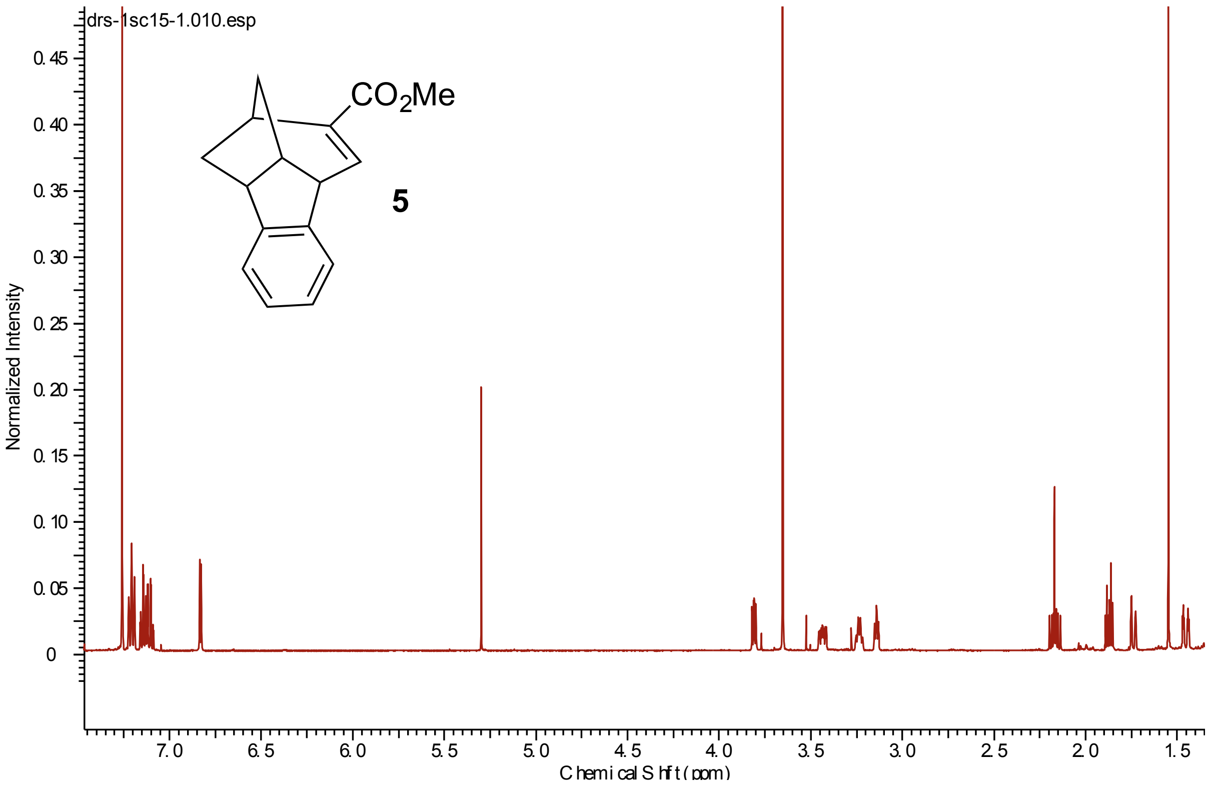
drs-1sc15-1.010.esp
CO2Me
5
Normalized Intensity
0.45
0.40
0.35
0.30
0.25
0.20
0.15
0.10
0.05
0
7.0
6.5
6.0
5.5
5.0
4.5
4.0
3.5
3.0
2.5
2.0
1.5
Chemical Shift (ppm)

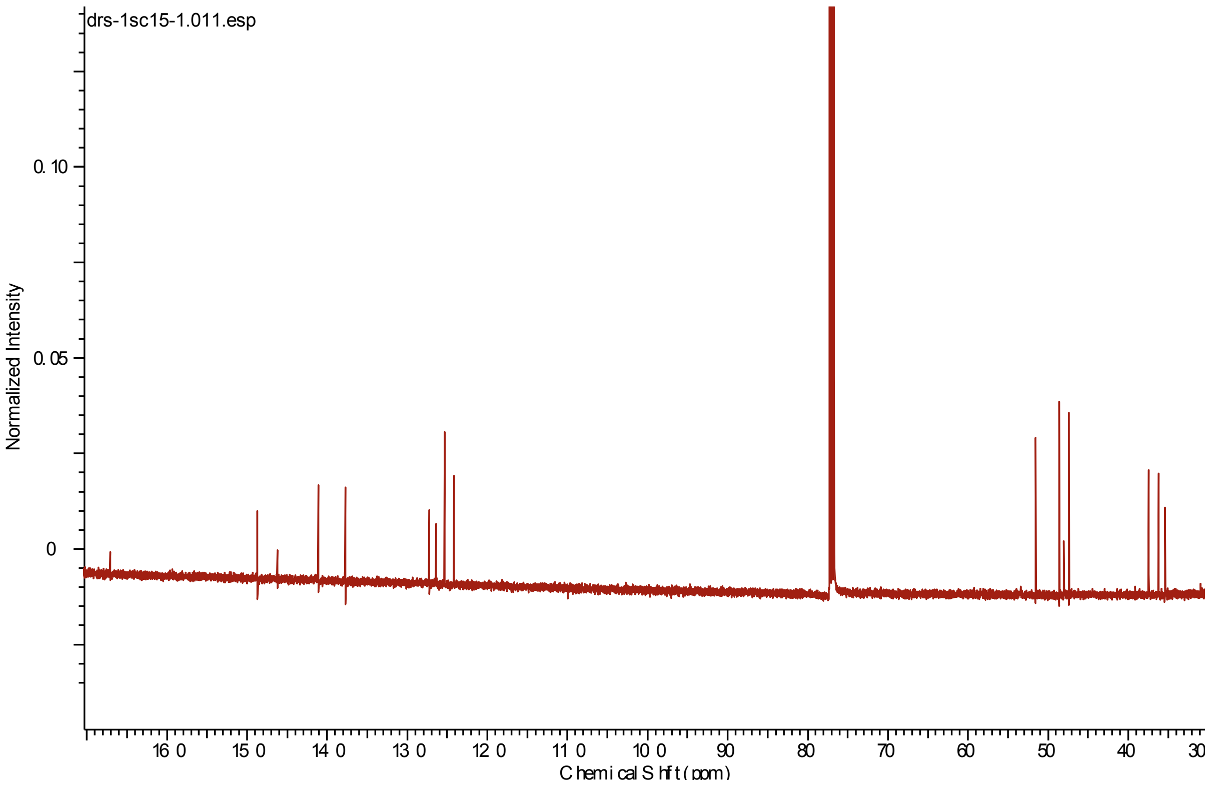
drs-1sc15-1.011.esp
Normalized Intensity
0.10
0.05
0
160
150
140
130
120
110
100
90
80
70
60
50
40
30
Chemical Shift (ppm)

X-ray Crystallographic Data:

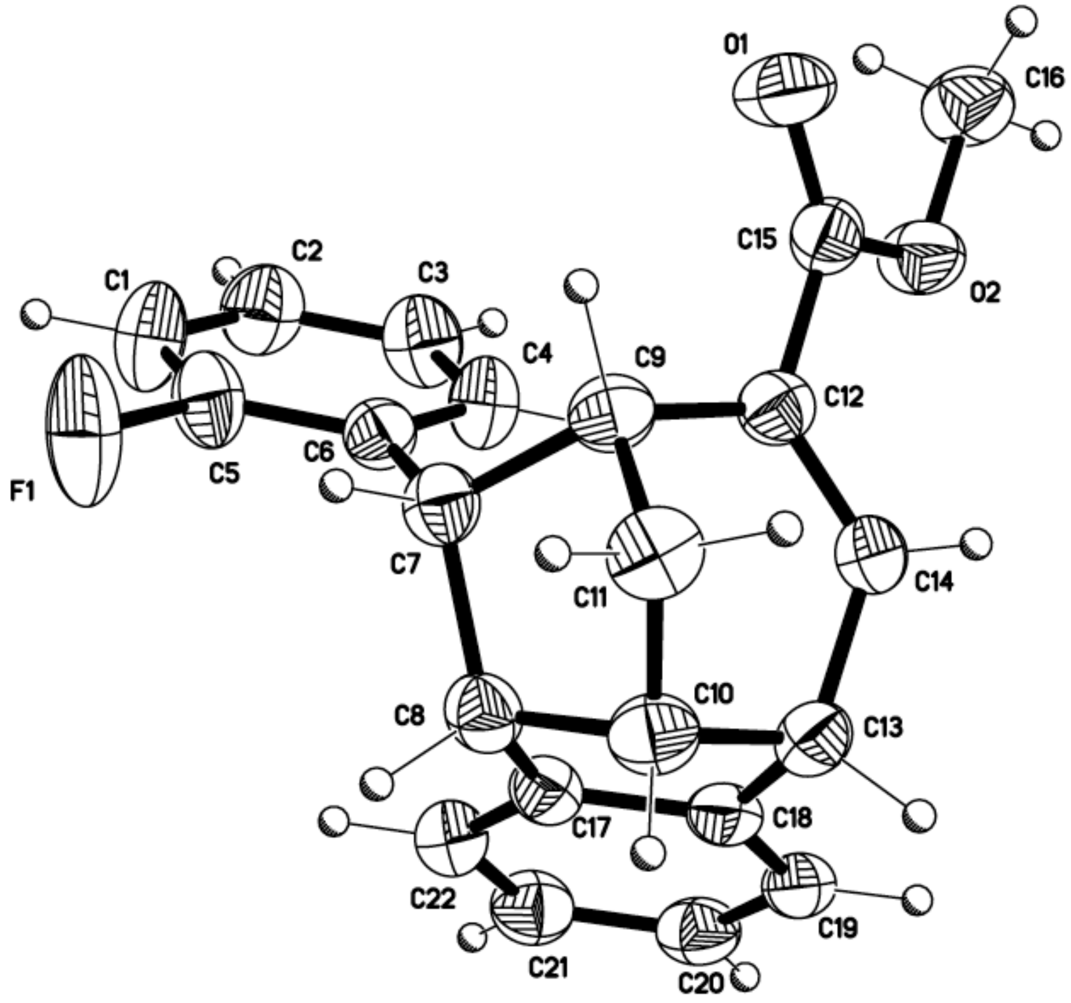

SI Figure 1. X-ray crystallographic image of **4d**.

SI Table 1. Crystal data and structure refinement for 4d.

Identification code	**4d**	
Empirical formula	C22 H19 F O2	
Formula weight	334.37	
Temperature	180(2) K	
Wavelength	0.71073 Å	
Crystal system	Monoclinic	
Space group	P2(1)/c	
Unit cell dimensions	a = 15.5436(6) Å	a= 90°.
	b = 8.7069(3) Å	b= 92.769(1)°.
	c = 12.5382(3) Å	g = 90°.
Volume	1694.9(1) $Å^3$	
Z	4	
Density (calculated)	1.310 Mg/m^3	
Absorption coefficient	0.090 mm^{-1}	
F(000)	704	
Crystal size	0.16 x 0.12 x 0.05 mm^3	
Theta range for data collection	3.52 to 27.47°.	
Index ranges	-14<=h<=20, -11<=k<=11, -15<=l<=15	
Reflections collected	9843	
Independent reflections	3836 [R(int) = 0.0345]	
Completeness to theta = 27.47°	98.7 %	
Absorption correction	Semi-empirical from equivalents	
Max. and min. transmission	1.000 and 0.928	
Refinement method	Full-matrix least-squares on F^2	
Data / restraints / parameters	3836 / 0 / 227	
Goodness-of-fit on F^2	0.906	
Final R indices [I>2sigma(I)]	R1 = 0.0423, wR2 = 0.0945	
R indices (all data)	R1 = 0.0782, wR2 = 0.1012	
Largest diff. peak and hole	0.216 and -0.241 e.$Å^{-3}$	

SI Table 2. Atomic coordinates (x 10^4) and equivalent isotropic displacement parameters ($Å^2$x 10^3) for **4d**. U(eq) is defined as one third of the trace of the orthogonalized U^{ij} tensor.

	x	y	z	U(eq)
C(1)	9492(1)	1585(2)	7204(1)	57(1)
O(1)	5847(1)	2492(1)	6390(1)	50(1)
O(2)	5565(1)	178(1)	7075(1)	44(1)
F(1)	9463(1)	1989(1)	5362(1)	75(1)
C(2)	9114(1)	1004(2)	8085(1)	52(1)
C(3)	8335(1)	276(2)	7968(1)	48(1)
C(4)	7928(1)	119(2)	6968(1)	43(1)
C(5)	9069(1)	1402(2)	6224(1)	44(1)
C(6)	8278(1)	708(2)	6053(1)	33(1)
C(7)	7857(1)	626(2)	4940(1)	35(1)
C(8)	7914(1)	-959(2)	4345(1)	37(1)
C(9)	6895(1)	1102(2)	4774(1)	36(1)
C(10)	7044(1)	-1074(2)	3690(1)	39(1)
C(11)	6715(1)	578(2)	3618(1)	42(1)
C(12)	6268(1)	255(2)	5454(1)	34(1)
C(13)	6477(1)	-2101(2)	4382(1)	37(1)
C(14)	6042(1)	-1189(2)	5218(1)	36(1)
C(15)	5880(1)	1111(2)	6327(1)	36(1)
C(16)	5192(1)	928(2)	7973(1)	50(1)
C(17)	7939(1)	-2402(2)	5010(1)	34(1)
C(18)	7137(1)	-3108(2)	4984(1)	34(1)
C(19)	7015(1)	-4491(2)	5498(1)	37(1)
C(20)	7715(1)	-5176(2)	6036(1)	41(1)
C(21)	8513(1)	-4472(2)	6071(1)	43(1)
C(22)	8634(1)	-3077(2)	5563(1)	41(1)

SI Table 3. Bond lengths [Å] and angles [°] for **4d**.

C(1)-C(2)	1.373(2)
C(1)-C(5)	1.375(2)
C(1)-H(1A)	0.9500
O(1)-C(15)	1.2068(16)
O(2)-C(15)	1.3508(16)
O(2)-C(16)	1.4465(16)
F(1)-C(5)	1.3676(15)
C(2)-C(3)	1.368(2)
C(2)-H(2)	0.9500
C(3)-C(4)	1.3831(19)
C(3)-H(3)	0.9500
C(4)-C(6)	1.3912(18)
C(4)-H(4)	0.9500
C(5)-C(6)	1.3763(19)
C(6)-C(7)	1.5150(18)
C(7)-C(9)	1.5561(19)
C(7)-C(8)	1.5728(19)
C(7)-H(7)	1.0000
C(8)-C(17)	1.5073(19)
C(8)-C(10)	1.552(2)
C(8)-H(8)	1.0000
C(9)-C(12)	1.5170(17)
C(9)-C(11)	1.5324(18)
C(9)-H(9)	1.0000
C(10)-C(11)	1.5278(19)
C(10)-C(13)	1.5500(19)
C(10)-H(10)	1.0000
C(11)-H(11A)	0.9900
C(11)-H(11B)	0.9900
C(12)-C(14)	1.3344(18)
C(12)-C(15)	1.4762(18)
C(13)-C(14)	1.5012(18)
C(13)-C(18)	1.5222(19)
C(13)-H(13)	1.0000
C(14)-H(14)	0.9500
C(16)-H(16A)	0.9800

C(16)-H(16B)	0.9800
C(16)-H(16C)	0.9800
C(17)-C(22)	1.3864(19)
C(17)-C(18)	1.3882(18)
C(18)-C(19)	1.3831(18)
C(19)-C(20)	1.387(2)
C(19)-H(19)	0.9500
C(20)-C(21)	1.383(2)
C(20)-H(20)	0.9500
C(21)-C(22)	1.388(2)
C(21)-H(21)	0.9500
C(22)-H(22)	0.9500

C(2)-C(1)-C(5)	118.08(14)
C(2)-C(1)-H(1A)	121.0
C(5)-C(1)-H(1A)	121.0
C(15)-O(2)-C(16)	116.17(11)
C(3)-C(2)-C(1)	119.71(14)
C(3)-C(2)-H(2)	120.1
C(1)-C(2)-H(2)	120.1
C(2)-C(3)-C(4)	120.44(13)
C(2)-C(3)-H(3)	119.8
C(4)-C(3)-H(3)	119.8
C(3)-C(4)-C(6)	122.04(13)
C(3)-C(4)-H(4)	119.0
C(6)-C(4)-H(4)	119.0
F(1)-C(5)-C(1)	116.71(13)
F(1)-C(5)-C(6)	118.15(12)
C(1)-C(5)-C(6)	125.12(13)
C(5)-C(6)-C(4)	114.56(12)
C(5)-C(6)-C(7)	120.45(11)
C(4)-C(6)-C(7)	124.99(12)
C(6)-C(7)-C(9)	118.69(10)
C(6)-C(7)-C(8)	116.52(10)
C(9)-C(7)-C(8)	104.22(10)
C(6)-C(7)-H(7)	105.4
C(9)-C(7)-H(7)	105.4
C(8)-C(7)-H(7)	105.4

C(17)-C(8)-C(10)	103.71(11)
C(17)-C(8)-C(7)	118.06(10)
C(10)-C(8)-C(7)	103.90(10)
C(17)-C(8)-H(8)	110.2
C(10)-C(8)-H(8)	110.2
C(7)-C(8)-H(8)	110.2
C(12)-C(9)-C(11)	107.13(11)
C(12)-C(9)-C(7)	115.81(10)
C(11)-C(9)-C(7)	100.22(10)
C(12)-C(9)-H(9)	111.0
C(11)-C(9)-H(9)	111.0
C(7)-C(9)-H(9)	111.0
C(11)-C(10)-C(13)	112.26(11)
C(11)-C(10)-C(8)	104.54(11)
C(13)-C(10)-C(8)	104.21(10)
C(11)-C(10)-H(10)	111.8
C(13)-C(10)-H(10)	111.8
C(8)-C(10)-H(10)	111.8
C(10)-C(11)-C(9)	100.29(10)
C(10)-C(11)-H(11A)	111.7
C(9)-C(11)-H(11A)	111.7
C(10)-C(11)-H(11B)	111.7
C(9)-C(11)-H(11B)	111.7
H(11A)-C(11)-H(11B)	109.5
C(14)-C(12)-C(15)	121.86(12)
C(14)-C(12)-C(9)	120.13(12)
C(15)-C(12)-C(9)	117.88(11)
C(14)-C(13)-C(18)	105.89(10)
C(14)-C(13)-C(10)	111.89(11)
C(18)-C(13)-C(10)	102.83(10)
C(14)-C(13)-H(13)	111.9
C(18)-C(13)-H(13)	111.9
C(10)-C(13)-H(13)	111.9
C(12)-C(14)-C(13)	122.07(13)
C(12)-C(14)-H(14)	119.0
C(13)-C(14)-H(14)	119.0
O(1)-C(15)-O(2)	122.45(12)
O(1)-C(15)-C(12)	124.84(12)

O(2)-C(15)-C(12)	112.70(12)
O(2)-C(16)-H(16A)	109.5
O(2)-C(16)-H(16B)	109.5
H(16A)-C(16)-H(16B)	109.5
O(2)-C(16)-H(16C)	109.5
H(16A)-C(16)-H(16C)	109.5
H(16B)-C(16)-H(16C)	109.5
C(22)-C(17)-C(18)	120.08(13)
C(22)-C(17)-C(8)	128.93(12)
C(18)-C(17)-C(8)	110.94(12)
C(19)-C(18)-C(17)	121.16(13)
C(19)-C(18)-C(13)	128.94(12)
C(17)-C(18)-C(13)	109.84(11)
C(18)-C(19)-C(20)	118.65(13)
C(18)-C(19)-H(19)	120.7
C(20)-C(19)-H(19)	120.7
C(21)-C(20)-C(19)	120.41(13)
C(21)-C(20)-H(20)	119.8
C(19)-C(20)-H(20)	119.8
C(20)-C(21)-C(22)	120.94(14)
C(20)-C(21)-H(21)	119.5
C(22)-C(21)-H(21)	119.5
C(17)-C(22)-C(21)	118.75(13)
C(17)-C(22)-H(22)	120.6
C(21)-C(22)-H(22)	120.6

Symmetry transformations used to generate equivalent atoms:

SI Table 4. Anisotropic displacement parameters ($Å^2$x 10^3) for **4d**. The anisotropic displacement factor exponent takes the form: $-2p^2[h^2 a^{*2}U^{11} + ... + 2 h k a^* b^* U^{12}]$

	U^{11}	U^{22}	U^{33}	U^{23}	U^{13}	U^{12}
C(1)	41(1)	70(1)	58(1)	8(1)	-8(1)	-18(1)
O(1)	66(1)	32(1)	53(1)	-6(1)	14(1)	0(1)
O(2)	53(1)	37(1)	42(1)	-3(1)	12(1)	-2(1)
F(1)	47(1)	119(1)	60(1)	30(1)	4(1)	-28(1)
C(2)	51(1)	60(1)	43(1)	-2(1)	-7(1)	-5(1)
C(3)	48(1)	62(1)	36(1)	8(1)	4(1)	-3(1)
C(4)	37(1)	50(1)	40(1)	8(1)	-1(1)	-9(1)
C(5)	38(1)	53(1)	43(1)	13(1)	7(1)	-5(1)
C(6)	34(1)	30(1)	36(1)	2(1)	3(1)	1(1)
C(7)	38(1)	35(1)	33(1)	5(1)	6(1)	-4(1)
C(8)	37(1)	39(1)	35(1)	2(1)	9(1)	-1(1)
C(9)	41(1)	31(1)	35(1)	2(1)	0(1)	2(1)
C(10)	46(1)	40(1)	32(1)	-3(1)	2(1)	1(1)
C(11)	49(1)	42(1)	35(1)	4(1)	-2(1)	1(1)
C(12)	34(1)	32(1)	35(1)	0(1)	-2(1)	2(1)
C(13)	36(1)	35(1)	39(1)	-6(1)	-2(1)	-3(1)
C(14)	29(1)	38(1)	40(1)	-2(1)	-1(1)	1(1)
C(15)	33(1)	34(1)	41(1)	0(1)	-2(1)	-2(1)
C(16)	61(1)	48(1)	40(1)	-7(1)	12(1)	-4(1)
C(17)	35(1)	34(1)	34(1)	-3(1)	7(1)	1(1)
C(18)	37(1)	32(1)	33(1)	-7(1)	6(1)	2(1)
C(19)	40(1)	32(1)	41(1)	-6(1)	10(1)	-3(1)
C(20)	54(1)	31(1)	40(1)	0(1)	10(1)	1(1)
C(21)	46(1)	39(1)	45(1)	1(1)	0(1)	7(1)
C(22)	36(1)	40(1)	45(1)	-3(1)	3(1)	-1(1)

SI Table 5. Hydrogen coordinates (x 10^4) and isotropic displacement parameters ($Å^2$x 10^3) for **4d**

	x	y	z	U(eq)
H(1A)	10032	2098	7269	68
H(2)	9392	1107	8773	62
H(3)	8071	-123	8577	58
H(4)	7393	-408	6904	51
H(7)	8184	1367	4503	43
H(8)	8403	-950	3856	44
H(9)	6825	2239	4842	43
H(10)	7116	-1528	2968	47
H(11A)	6093	620	3410	50
H(11B)	7040	1196	3111	50
H(13)	6056	-2723	3935	44
H(14)	5592	-1654	5591	43
H(16A)	4894	166	8396	74
H(16B)	4781	1711	7712	74
H(16C)	5650	1416	8419	74
H(19)	6463	-4963	5482	45
H(20)	7646	-6136	6382	50
H(21)	8986	-4949	6448	52
H(22)	9183	-2595	5593	49

Biological Methods:

High Content Analysis – Mitotic Index

HCA was performed using an Arrayscan II HCS reader and integrated software from Cellomics. U2oS osteosarcoma cells (ATCC) were seeded in a NUNC clear flat-bottomed 96-well plate at 10,000/well in a total of 100 µL. They were incubated at 37 ºC overnight. Cells were then treated with compounds (25 µL) to give the appropriate concentration. Cells were then incubated at 37 ºC for 20 h. 50 µL 12.5 % formaldehyde was added to each well. This was incubated at RT for 10 min, before the formaldehyde was gently removed. 100 µL/well permeabilization buffer (PB, contains PBS + 0.1% Triton X-100), was then added to each well, incubating for 10 min. PB was removed and wells were washed with 100 µL/well blocking buffer (BB, contains PBS + 1% BSA). BB was removed and 50 µL/well of primary antibody solution (anti-pH3 (S10), Abcam, ab5176, 1:2000) was added. Plates were incubated for 1 h at room temperature. The antibody was removed and wells washed with 2 x 100 µL/well BB. BB was removed and 50 µL/well of secondary antibody solution containing Hoechst (Invitrogen, H3570, 1:2500) and AlexaFluor 488 Goat anti-rabbit IgG (Invitrogen, A11034) was added. Plates were incubated at RT for 1 h in the dark. Secondary antibody solution was removed and plates washed with 2 x 100 µL BB. The BB was then removed and 100 µL PBS/well were added. The plates were sealed with opaque film and images taken on a 20x 0.4 NA objective. Data was analysed on Cellomics Arrayscan software using the Target Activation v4 protocol. Critical output features are: ValidObjectCount and %Responder_AvgIntenCh2. EC_{50} data was calculated using Prism (Graphpad) and is an average of three independent experiments conducted in triplicate.

Sulforhodamine B colorimetric assay for cytotoxicity screening

U2oS cells were seeded at 4000 cells/well in 180 µL and incubated for 20 h at 37 °C. Compounds were then added in 20 µL to give the desired concentrations. After incubation for 72 h at 37 °C the medium was removed by aspiration, and 100 µL of 1% TCA solution was added. This was incubated for 1 h and then removed. The plates were washed 4 x with tap water and the plates were allowed to air dry at RT. 100 µL of a 0.057 % wt/vol solution of Sulforhodamine B (Sigma Aldrich) were added to each well, incubating for 30 min. The plates were then washed quickly with 4 x 100 µL of acetic acid solution (1%) and then air-dried. 200 µL of TRIS buffer (10 mM, pH

8.0) was added to each well to resolubilise the dye. The plates were then read at 510 nm on a Infinite® M200 plate reader (Tecan, Austria). GI_{50} data was calculated using Prism (Graphpad) and is an average of three independent experiments conducted in triplicate.

Confocal Microscopy

200,000 U2oS cells were seeded on coverslips in 2 mL medium in a six-well plate and incubated at 37 °C overnight. Compounds were added in DMSO to the appropriate concentration and the cells were incubated for 16 hours. The medium was then aspirated and the cells were then fixed in 1 mL PBS containing 4% *para*-formaldehyde for 10 min. Cells were permeabilised in PBS containing 0.1% Triton-X (PBS-T) for 10 min and then washed with 1 mL PBS containing 1% BSA (PBS-BSA). Tubulin was visualized using an α-tubulin antibody (1:1000) in 500 µL for 2 h. The primary antibody was then removed and the cells were washed with 1 mL PBS-BSA three times. Alexafluor-488 conjugated goat anti-rabbit (Invitrogen, A11034) secondary antibody was then added in 500 µL PBS-BSA for 1 h. The secondary antibody was removed and cells were washed twice with PBS-BSA and once with PBS. The coverslips were mounted on slides with mounting medium containing DAPI and imaged on a Zeiss LSM-510 confocal microscope with a 100X objective.

High Content Analysis – Apoptosis:

HCA was performed using an Arrayscan II HCS reader and integrated software from Cellomics. U2oS cells were seeded in a NUNC clear flat bottomed 96-well plate at 4000/well in a total of 180 µL. They were incubated at 37 ºC overnight. Cells were then treated with compounds (20 µL at 10X concentration) to give the final desired concentration. Cells were then incubated at 37 ºC for 72 h. The medium was gently removed from all the wells and 100 µL cold methanol was added to each well. This was incubated at -20 ºC for 3 min, before the methanol was removed. The wells were washed for 3 x 5 min in 100 µL/well permeabilization buffer (PB, contains PBS + 0.1% Triton X-100). PB was removed and wells washed with 100 µL/well blocking buffer (BB, contains PBS + 1% BSA). BB was removed and 50 µL/well of primary antibody solution (anti c-PARP, 1:800) was added. Plates were incubated for 2 h at room temperature. The antibody was removed and wells washed with 2 x 100 µL/well BB. BB was removed and 50 µL/well of secondary antibody solution containing Hoechst (1:2500) and AlexaFluor 488 Goat anti-mouse IgG (1:500) was added. Plates were incubated at RT for 1 h in the dark. Secondary antibody solution was

removed and plates washed with 2 x 100 µL BB. The BB was then removed and 100 µL PBS/well were added. The plates were sealed with opaque film and images taken on a 20x 0.4 NA objective. Data was analysed on Cellomics Arrayscan software using the Target Activation v4 protocol. Critical output features are: ValidObjectCount and %Responder_AvgIntenCh2.
